# A review of recent advances in data-driven computer vision methods for structural damage evaluation: algorithms, applications, challenges, and future opportunities

**DOI:** 10.1007/s11831-025-10279-8

**Published:** 2025-04-03

**Authors:** Xiao Pan, Tony T. Y. Yang, Jun Li, Carlos Ventura, Christian Málaga-Chuquitaype, Chaobin Li, Ray Kai Leung Su, Svetlana Brzev

**Affiliations:** 1 Department of Civil and Environmental Engineering, The Hong Kong University of Science and Technology, Clear Water Bay, Hong Kong; 2 Department of Civil Engineering, The University of British Columbia, Vancouver, Canada; 3 Centre for Infrastructural Monitoring and Protection, Curtin University, Perth, Australia; 4 Department of Civil and Environmental Engineering, Imperial College London, London, UK; 5 Department of Civil Engineering, The University of Hong Kong, Hong Kong, China

**Keywords:** Structural damage detection, Structural health monitoring, Computer vision, Image processing, Machine learning, Deep learning, Sensor fusion, Unmanned aerial and ground vehicles

## Abstract

Computer vision techniques have gained great traction in civil infrastructure inspection and monitoring. This paper conducted a systematic review of recent data-driven computer vision algorithms in structural damage detection published during the past 5 years. The theories of prevalent computer vision models are first reviewed with an emphasis on the progressive innovation in algorithms’ architecture. Then, recent applications of computer vision models for structural damage evaluation are discussed, which are classified into different structural categories by their material types (i.e., concrete, steel, masonry, timber) at three hierarchical levels including damage recognition, localization, and quantification. In particular, the paper also highlights the current state of using computer vision for damage assessment of timber structures, which remains under-explored compared to concrete and steel structures. Next, the paper scrutinizes existing structural damage inspection guidelines to identify key technological gaps between the capability of existing computer vision methods and manual inspection practices in the field. Finally, the paper summarizes existing challenges and recommends future research opportunities including the integration of computer vision methods with multimodal large language models, sensor-fusion, and mobile inspection approaches.

## Introduction

Civil engineering structures such as buildings and bridges deteriorate continuously due to aging and environmental impacts. In North America, the 2021 Report Card for America’s Infrastructure [1] shows the infrastructure scores a GPA of “C−”. The long-term infrastructure investment gap for inspection and maintenance in the US was reported to be $2.59 trillion over 10 years. On the other hand, the Canadian Infrastructure Report notes that 40% of the infrastructure is rated in poor condition, with estimated repair or replacement costs of $141 billion [2]. Regions such as the British Columbia province of Canada are situated in a high seismic zone, where a significant earthquake can result in $75 billion in financial losses and thousands of casualties and injuries [3, 4]. Failure to early identify the deteriorating infrastructure may cause catastrophic results, which can significantly hinder regional resilience against manmade and natural hazards, and further impact the social and economic development. Traditional infrastructure inspection relies on human inspectors, which is inefficient, inherently biased, and heavily dependent on the proper training and experience of inspectors. On the other hand, as civil infrastructure is relatively large and can be constructed in a remote and harsh environment, visual manual inspection can be inconvenient and unsafe for inspectors. These issues can be further exacerbated by a significant labor shortage of skilled workers in some developed countries such as Canada, which results in very high long-term maintenance costs and becomes even more challenging right after the event of natural hazards such as earthquakes, hurricanes, flooding, and wildfires.

Over the past decades, structural health monitoring technologies have been developed to enhance the efficiency, capability, and convenience for infrastructure inspection and monitoring, such as vibration-based Structural Health Monitoring (SHM), and non-destructive testing and evaluation (NDTE)-based SHM. While the vibration-based and NDTE-based methods have made remarkable achievements, there exist several limitations, such as complex instrumentation procedures, high cost, and sensitivity of sensor performance to environmental conditions such as temperature and humidity, to name a few. On the other hand, in recent years, with the rapid development and open-source nature of algorithms in the computer vision (CV) community, and the knowledge and technology transfer rapidly from the CV community into the civil and structural engineering field. As a result, CV-based SHM methods have evolved into an efficient, economical, and accurate complimentary approach to other SHM methods. These CV methods have been investigated for many different structural engineering applications such as structural system or component recognition, structural damage detection, structural vibration and deformation measurement, structural experimental testing, etc. This paper is focused on reviewing the recent advances in CV methods for structural damage detection. There are several related review papers published. For example, Feng and Feng [5] reviewed CV-based SHM method with a strong focus on CV-based vibration measurement to assist the vibration-based SHM. Spencer et al. [6] presented a review of CV-based SHM with a broad focus, including structural component recognition, damage detection, and long-term monitoring solutions. Dong and Catbas [7] reviewed both the system-level and component-level CV-based SHM methods. Di Mucci et al. [8] reviewed various AI methods for SHM applications, where the AI-driven CV methods were discussed including image classification, object detection, segmentation and feature detection. Guo et al.[9] reviewed image-based surface defect detection from the data perspective, where a novel data management framework was proposed to improve the performance of defect detection models from three aspects, including data collection, data pre-processing, and data-driven training. In general, these studies concluded that major challenges for CV methods include requirements of extensive annotation, inconsistent labelling for complex topology of defects, lack of training data, data imbalance, negative environmental effects, and limitations of data collection equipment, etc. Despite the promising outcomes, these studies had a relatively broad or different focus compared to the present paper which is targeted at identifying the key technological gap between the existing CV-based structural damage inspection and manual visual inspection in the field, and recommending possible future directions accordingly. Besides, many former review articles were primarily focused on reinforced concrete (RC) and steel structures, while much less on masonry and timber structures. Furthermore, there exist key advances in CV-based damage detection since the publication of the former review papers, as will be discussed in Sects. 3 and 4. Therefore, it is crucial to provide readers and newcomers with the knowledge and applications of state-of-the-art algorithms, requirements of existing guidelines, as well as contemporary trends.

Within this context, this paper presents a comprehensive review of recent advances in data-driven computer vision methods for structural damage detection (SDD). The objectives, novelties and main contributions of the present paper include:

- This paper provides an in-depth and comprehensive review on data-driven computer vision-based structural damage assessment, including detailed discussion of algorithm architecture, engineering applications, existing challenges, and future opportunities, to provide key theories and engineering case studies for readers to quickly grasp the fundamental knowledge and state-of-the-art development of the related research.
- It presents a comprehensive review of the latest convolutional neural network (CNN) and Transformer-empowered computer vision methods for SDD published during the past five years. An in-depth review of related research on concrete, steel, masonry, and timber structures at three hierarchical levels is presented, including damage recognition, damage localization, and damage quantification. It is generally recognized that machine learning is a rapidly emerging field compared to traditional engineering fields. As structural health monitoring (SHM) is multidisciplinary in nature, it requires the exploration of coherent, latest, and most effective machine learning and data-driven algorithms to tackle challenging domain-specific problems.
- It has scrutinized the computer vision algorithms’ capability and their compliance with multiple existing inspection guidelines to identify key challenges and technological gap between the current research and manual field practices.
- The paper outlined three promising future research directions including the development of prevalent multimodal Vision-Language Models (VLMs), CV-enhanced multi-sensor fusion, and efficient deployment of CV methods with mobile equipment.

The rest of the paper is organized as follows. Section 2 describes the selection and screening criteria for the related publications. Section 3 reviews the basics and architecture of prevalent CV methods for SDD research, with a focus on recent data-driven machine learning models. The section intends to provide readers (particularly newcomers in the related field) with basic knowledge of recent successful CV models used in SDD applications, by highlighting the main features and progressive improvements of these models. Section 4 reviews the applications of CV-based methods for structural damage evaluation, which are classified into damage recognition, localization, and quantification, for multiple damage types across multiple structural categories (i.e., concrete, steel, masonry, timber). Section 5 reviews existing structural inspection guidelines, and identifies the technological gap of the CV methods with reference to these guidelines. Section 6 outlines three future research directions to address the existing challenges, where preliminary research findings including the authors’ ongoing work and other related studies are discussed. Section 7 concludes the main findings.

## Methodology

**Fig. 1:**
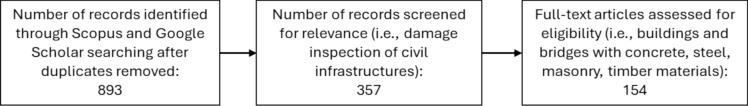
Manuscript screening process using the PRISMA approach

To find the related articles, Scopus and Google Scholar have been selected as the search engines as they are generally recognized to provide a comprehensive database for academic publications which cover almost all the high-quality papers indexed by the Web of Science. Searching keywords such as “image-based”, “vision-based”, “computer vision”, “structural damage”, “damage detection”, “health monitoring”, “inspection”, “assessment”, “machine learning”, “deep learning”, “attention mechanism”, “transformer”, etc., were used, together with the “AND” and “OR” operators to form the search combo strings. The year range was set to the past 5 years at the time of search (i.e., around August 2024). The search outcomes from Scopus and Google Scholar were merged while eliminating the duplicate literature, which yielded 890 publications. Next, a manual screening process was conducted with the following criteria: (1) Peer-reviewed journal articles are prioritized, while only a few conference proceedings and book chapters closely related to the topic and discussion are included. This is because journal articles generally go through a more rigorous review process. Also, many conference papers report preliminary findings or summarize partially completed work from the corresponding journal papers. Therefore, the review quality will not be compromised by prioritizing the referred journal articles. This yields about 350 papers. (2) To limit the scope, this review paper focuses on reviewing civil infrastructure primarily in the field of structural engineering, with a focus on buildings and bridges. Other infrastructure in geotechnical and transportation engineering (e.g., roads, railway tracks, tunnels, dams, underground pipelines) is excluded in the present paper. Only the papers published in the English language are reviewed. After the second screening criterion, about 160 papers were selected. The procedures are illustrated in Fig. 1. In addition, the primary journal sources of the selected papers are summarized in Table 1. It can be observed that Automation in Construction, Computer-Aided Civil and Infrastructure Engineering, Structural Health Monitoring are the top three journals that publish the most articles in the related topic. The geographic zones of the selected papers were obtained through co-authorship and country analysis in VOSviewer, which are presented in Table 2.

**Table 1:** A list of selected journals with the number of publications (with at least three selected papers)

Automation in Construction	26
Computer-Aided Civil and Infrastructure Engineering	24
Structural Health Monitoring	21
Sensors	15
Construction and building materials	10
Structural Control and Health Monitoring	8
Engineering Structures	8
Applied Sciences	7
Journal of Building Engineering	6
Journal of Civil Structural Health Monitoring	6
Remote sensing	6
Smart Structures and Systems	4

**Table 2:** A list of countries with the number of publications (with at least three selected papers)

P.R. China	57
USA	32
South Korea	26
Canada	21
Australia	14
Japan	10
England	6

This review is focused on discussing the model architecture, applications, guidelines, challenges, and future opportunities related to the recent CV-based SDD studies mainly published during the past five years. For former related research prior to 2019, readers are recommended to visit Spencer et al. [6] and Dong and Catbas [7]. In the meantime, this paper excludes a detailed review of CV-based structural vibration measurement, or CV-based modal identification, as it falls into the service of vibration-based SHM applications. Interested readers are referred to other relevant existing literature [5, 7, 10–15] which provide a detailed discussion of hardware setup and software algorithms for these applications.

## Basics and architectures of CV algorithms for SDD

Prior to the year of 2012, most CV methods developed for SDD were based on traditional image processing techniques (IPT) such as color thresholding, image filtering, template matching, edge-based segmentation, threshold-based segmentation, region-based segmentation, histogram transform, and texture pattern recognition [16]. For example, Sinha et al. [17] investigates different types of filtering techniques, feature extraction, and pattern recognition algorithms in identifying underground pipeline defects such as cracks and holes. It was concluded that the background lighting condition and the complicated texture on the pipe surface impose a big challenge for the damage detection process. German et al. [18] employed an entropy-based thresholding algorithm to localize the spalling area of RC columns. Further, the authors employed a connected image pixel labelling algorithm, a global adaptive thresholding algorithm, and a template matching algorithm, to estimate the concrete spalling length and depth. The spalling localization accuracy was reported ~80%, which is much lower than the current state-of-the-art (SOTA) models.

The transition from traditional methods to data-driven methods occurred when the breakthrough was achieved by AlexNet [19], which is built upon the CNN architecture. AlexNet shows substantial accuracy increase and robustness in image classification, object detection, and semantic segmentation tasks compared to former non-data-driven image processing algorithms. With the success of AlexNet, CNNs have gained great traction and further development in various image processing tasks such as image classification, object detection, instance or semantic segmentation, and visual object tracking. Specifically, other deeper CNN networks such as VGG Net [20], Inception [21], Deep Residual Net [22], Xception [23], DenseNet [24], MobileNet [25], SENet [26], ECA-Net [27], and Coordinate attention-based Networks [28], etc., have been developed. The main SDD tasks and the corresponding CV algorithms are summarized in Table 3.

**Table 3:** Summary of CV algorithms for SDD

SDD tasks	Damage classification	Damage localization	Damage quantification
CV algorithms	Image classification	Bounding box detection	Pixel-based quantification
		Pixel-wise segmentation	Point cloud-based quantification

This section intends to provide a review of the theoretical aspect of the prevalent data-driven CV methods for SDD published roughly between late 2019 and 2024. Specifically, these methods are primarily built upon the CNNs, with more recent studies attempting to investigate attention mechanism or Vision Transformer (ViT) architecture. It should be noted that this section is focused on reviewing the prevalent machine learning-based CV methods including their critical contributions, potential limitations, and progressive improvements. Regarding a detailed step-by-step network design guide, training settings, and pre-training and fine-tuning tricks for different machine learning models, readers are encouraged to visit the respective original research papers.

### CV Algorithms for Damage Classification

**Fig. 2:**
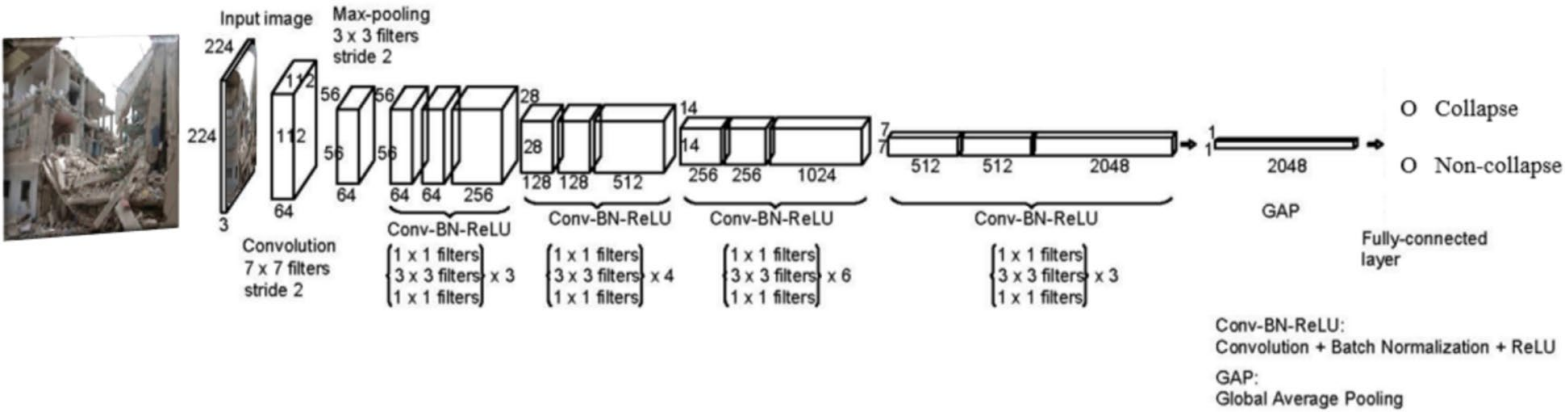
Vision-based collapse identification using a CNN-based classification model

The first fundamental task is image classification, which in the case of CV-based SDD, involves the visual recognition of structural damages from images. This may include identification of building collapse, classification of structural failure modes (e.g., flexural, shear, combined flexural and shear mode), or damage state (light, moderate, severe, etc.) as defined in existing design codes and assessment guidelines. Figure 2 depicts the architecture and application of a promising CNN model, ResNet (which receives over 250,000 citations in Google Scholar), for damage classification. In general, a CNN model typically consists of a series of layers, including the input layer, convolution (Conv) layers, rectified linear unit (ReLU) layers, pooling layers, fully connected (FC) layers, and the output layer (e.g., Softmax layer). The input layer takes images of any shapes and resizes them to the dimension required by the CNN model. The Conv layer followed by the subsequent layer, ReLU, constitutes the essential computational block (Conv-ReLU) for CNN models, which is the key difference between CNNs and traditional feedforward fully connected neural networks. Pooling layers are used to pool features from the preceding layers, while also reducing the size of feature maps to lower the number of parameters to learn and consequently the computational cost. FC layers are generally placed right before the output layer to flatten the feature maps into a single vector, which will be used by the post-layers for further processing such as calculation of optimization losses. The output layer generates the network output, such as class prediction or object location. In summary, compared to the traditional feedforward fully connected neural networks, the main advantage of CNNs is the number of learnable parameters is significantly less, leading to much higher computational efficiency. In addition, the spatial locality of pixel dependencies is preserved in CNN models, and the trained filters are enforced to provide the strongest response to local patch features.

As will be discussed in Sect. 4 in detail, Numerous SOTA CNN models have been investigated in SDD research in recent years. In summary, research has shown that the use of formerly developed models (e.g., VGG, ResNet) can achieve comparative and satisfactory results with SOTA models, with proper training setting and parameter fine-tuning. This may be partly attributed to the fact that a vast majority of existing SDD research only needs to consider a small number of class labels during training and testing (i.e., a limited number of damage types on a limited number of structural components in a single paper), which is much less than that of other CV competition datasets such as ImageNet which has 1000 categories, creating more challenges to be addressed by a deeper and novel network architecture as seen in the SOTA models. Therefore, to date, earlier developed models such as ResNet are still popular as a backbone network for a wide range of SDD applications. The pros and cons of major CV algorithms for SDD are summarized in Table 4.

**Table 4:** Summary of CV algorithms for damage classification

Major CV algorithms for damage classification	Advantages	Disadvantages
Machine learning models, CNN models, Attention-based and Transformer models, etc.	Rapid assessment of damage conditions at the system level and component level	Limited to damage recognition only, without knowing the damage locations and damage dimensions

### CV Algorithms for Damage Localization

**Fig. 3:**
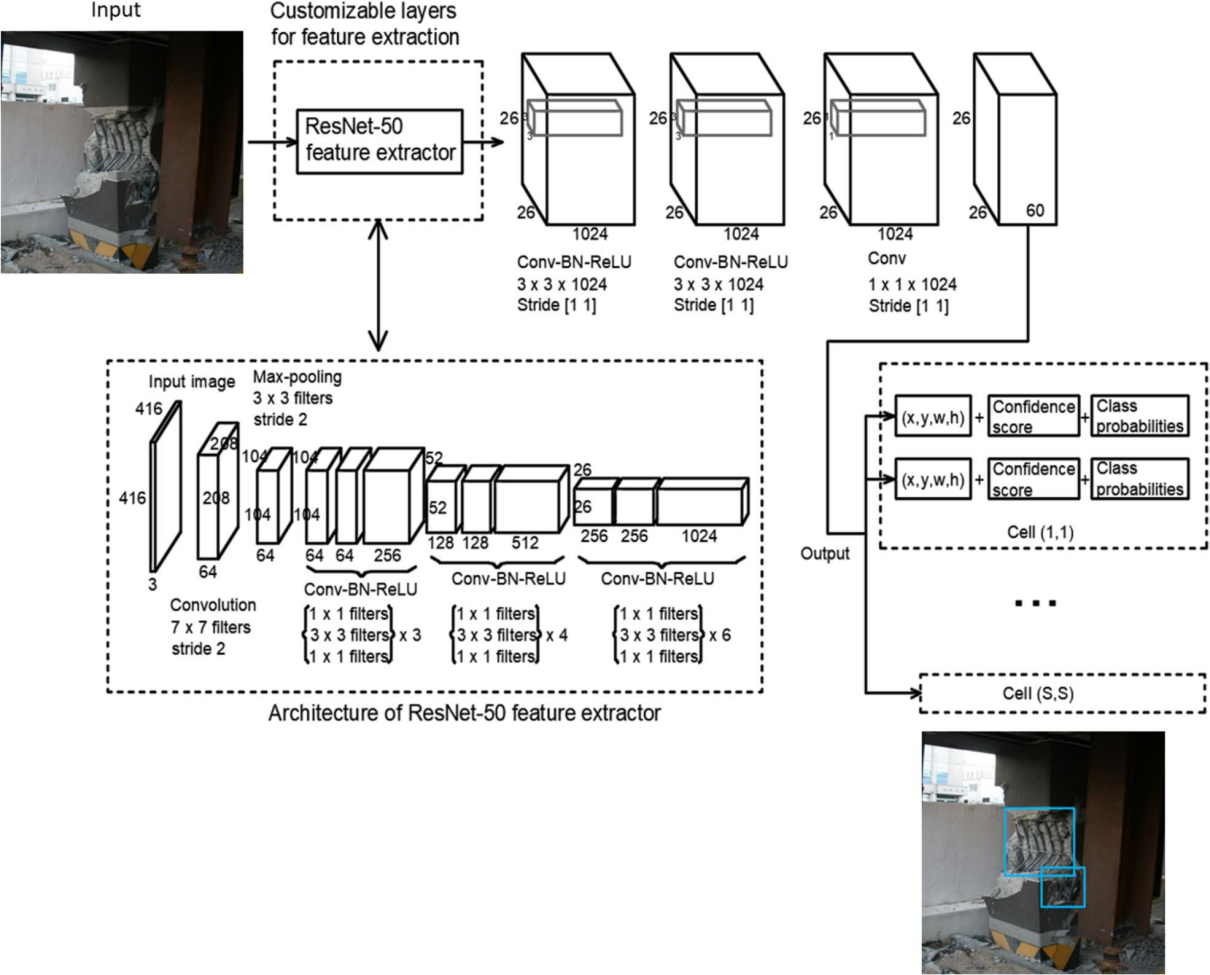
Vision-based localization of steel reinforcement exposure using bounding boxes

**Fig. 4:**
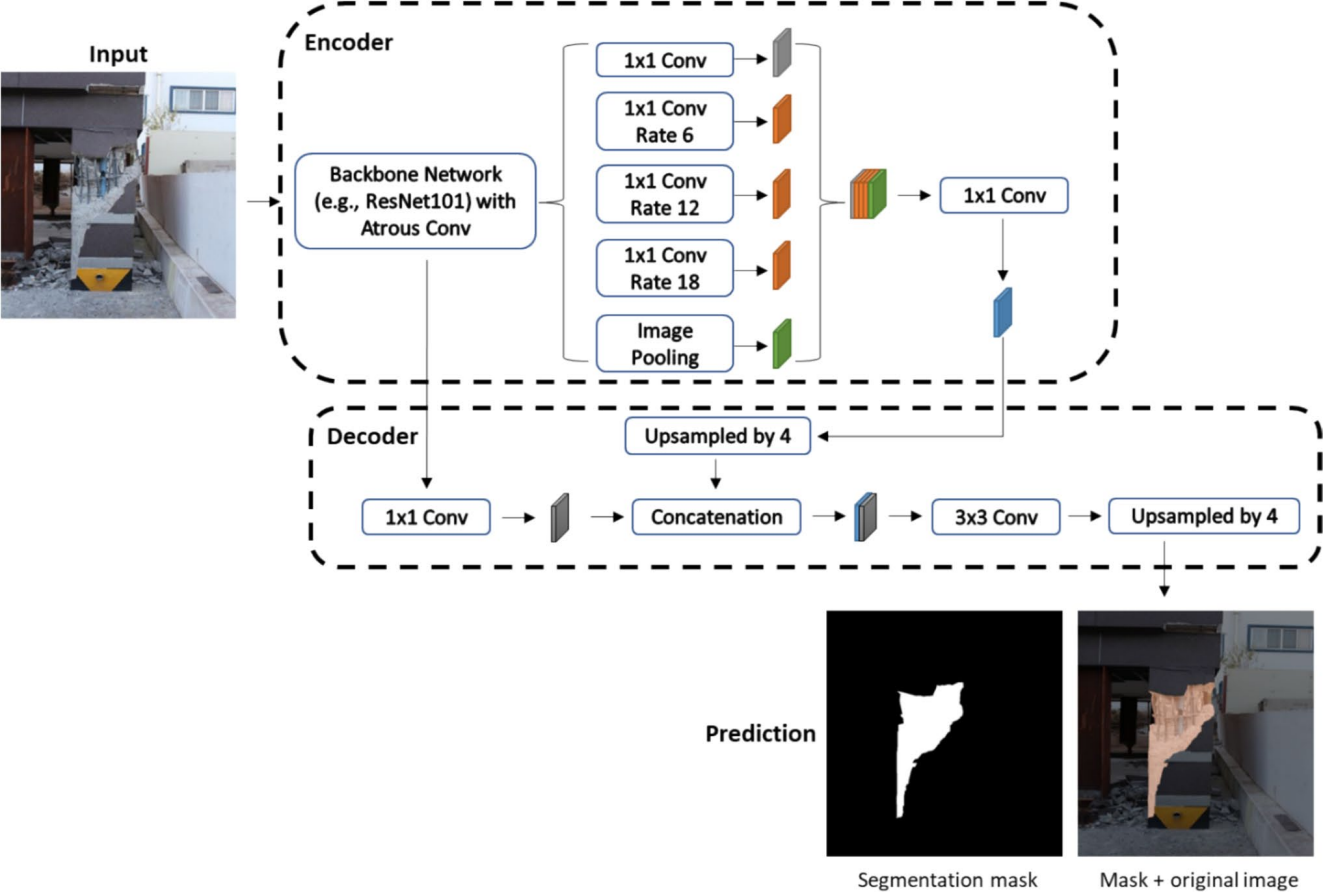
Localization of concrete spalling using pixel-wise masks

The second fundamental CV task is object detection, which in SDD research, pertains to the localization of damage features (e.g., cracks, spalling) or damaged portions (e.g., mid-span, connections) of structural components. The CV-based localization methods can be divided into one-stage and two-stage methods. Earlier research generally employs two-stage methods [7], where the sliding window technique or image partition is performed to generate small image patches, which will then be processed by a classification model or image binarization and thresholding to achieve localization goals. More recently, one-stage methods have prevailed, especially for SDD research published during the past five years, because they offer several advantages such as end-to-end training and deployment, high accuracy, high efficiency, and high robustness. These one-stage CV-based localization methods can be achieved using bounding box prediction or pixel-wise segmentation. Figure 3 shows an example of one-stage CV-based localization of the steel reinforcement exposure using bounding boxes detection models [29], while Fig. 4 shows CV-based localization of concrete spalling using pixel-level segmentation models [30]. As this paper is focused on reviewing the literature published in the last 5 years, the architecture of related and predominant one-stage CV methods for damage localization will be discussed in this section. The pros and cons of the CV algorithms for damage localization are summarized in Table 5.

**Table 5:** Summary of CV algorithms for damage localization

Major CV algorithms for damage localization	Advantages	Disadvantages
Bounding box-based detection models (e.g., RCNN, SSD, YOLO, etc.)	Relatively fast speed compared to pixel-wise segmentation models	Localization of damage is achieved by bounding boxes, which is not very accurate and limited to 2D space
Pixel-wise segmentation models (FCN, Mask R-CNN, U-net, DeepLab, Vision Transformer, etc.)	Localization of damage is achieved by pixel masks, which is more accurate than bounding box models	Relatively slow speed compared to bounding box models. Localization of damage is limited to 2D space

#### Bounding box detection models

Bounding box detection models can be broadly classified as single-class object detectors and multi-class object detectors. The main differences between a single-class and multi-class detector are single-class detectors generally use only a regression layer head to predict bounding boxes of a single class, while a multi-class object detector may use a fully-connected layer head, including a regression layer and SoftMax classifier, to localize and distinguish different object classes in an image. From the model architecture’s perspective, bounding box detection models can also be categorized into one-stage object detectors and multi-stage object detectors, as detailed in the sections below.

##### R-CNN, Fast R-CNN, Faster R-CNN

The first promising object detection model built upon the CNN architecture is R-CNN [31]. In the first step, the R-CNN object detector employs region proposal algorithms such as Edge Boxes [32] to generate preliminary regions-of-interest (ROIs) on an input image. Then, the ROIs are cropped out from the original images to form small image patches, which will then be processed by a backbone CNN model (e.g., VGG) to predict a class for each image patch. In the meantime, the final predicted bounding boxes (i.e., locations of objects in an image) are generated by refining the ROI proposals using a class-specific support vector machine (SVM) which has been trained on the extracted features by the backbone CNN.

Later, to enhance the efficiency of the R-CNN model, Fast R-CNN [33] was developed. In the R-CNN model, the region proposal algorithm generates numerous ROIs which inevitably have many overlaps with each other. Consequently, the overlapped image patches are processed multiple times by the CNN backbone to generate the corresponding feature maps, leading to significant and extra computation costs. Therefore, the main contribution of Fast R-CNN is to address this issue. First, similar to the R-CNN model, the Fast R-CNN employs a predefined region proposal function such as Edge Boxes to generate ROIs. In the meantime, the Fast R-CNN takes the entire image as the input and processes it only once, as opposed to R-CNN which processes small image patches many times. Further, the Fast R-CNN uses the concept of ROI pool to extract CNN features with respect to each region proposal, followed by classification and bounding box regression. In summary, as the Fast R-CNN shares the computation of the overlapped regions by processing the entire image, the efficiency of the Fast-RCNN is greatly enhanced over the R-CNN.

Furthermore, having realized that traditional region proposal functions can generate numerous irrelevant ROIs with respect to the ground truth bounding boxes, the Faster R-CNN model [34] was developed, which introduced a region proposal network (RPN) to generate region proposals inside the object detection model. The advantages of RPN include, (1) it can be trained with other network modules (i.e., backbone CNN, bounding box regression) within an end-to-end network scheme, (2) it can generate more relevant ROIs tuned to the data because the RPN has been trained exclusively on them, and (3) once being trained, it is generally more efficient than traditional region proposal functions such as Edge Boxes during deployment. Research has shown that the Fast R-CNN model achieves about real-time performance. Apart from the RPN, the remaining architecture of the Faster R-CNN model is similar to that of the Fast R-CNN.

In summary, R-CNN, Fast R-CNN, and Faster R-CNN are called multi-stage object detectors as they include region proposal generation, feature map generation, classification, and bounding box regression at multiple stages.

##### Single Shot Detector (SSD)

Compared to multi-stage object detectors such as R-CNN and its incremental variants, SSD developed by Liu et al. [35], is a single-stage detector with an extremely simple and straightforward architecture that provides fast real-time performance and easy end-to-end training. The SSD model has two main components including a backbone network and the SSD detection head. The backbone can be from any pre-trained classification models (e.g., VGG, ResNet) by removing the final few fully connected layers. The SSD detection head simply consists of a series of Conv blocks which output bounding boxes and class predictions. The SSD employs small Conv filters at multiple feature maps to detect objects at different scales. The network associates a fixed set of boxes with each grid cell, leading to a final output size of (c + 4) × k × m × n, where m × n is the feature map size, c is the number of classes, 4 is the predicted bounding box offsets, k is the number of boxes associated to each grid. The main limitations of SSD are: (1) it is not as accurate as the R-CNN variants which can take advantage of multiple networks. (2) as it associates a fixed set of boxes to each grid cell, meaning its generalizability can be worse in detecting objects at very different scales than those in the training set.

##### You Only Look Once (YOLO) models

Redmon et al. [36] propose a single-stage regression-based CNN object detector, YOLO, which reasons straight from image pixels towards bounding box locations and class probabilities. The name, YOLO, comes from the fact that the model only looks once at an image in order to make a prediction. This network design leads to a relatively simple architecture and real-time speed. First, YOLO divides the input image into a S × S grid pattern (e.g., S = 7 in the first demonstration [36]). An object is assigned to the grid cell within which the center of the object appears. If the center of an object falls into a grid cell, that grid cell is responsible for detecting that object (we assign the object to the grid cell where the center of the object exists). The architecture of YOLO is rather straightforward, which consists of an input layer, standard Conv, pooling, FC layers, and an output layer. The shape of the final output tensor is S × S × (B × 5 + C), where S is the grid number, B is the number of bounding boxes, C is the number of classes, and the number “5” comes from the sum of 4 bounding box location parameters and 1 confidence score (objectness). For example, in Redmon et al. [36], 7 grids and 2 bounding boxes are used, leading to the final output shape of 7 × 7 × (2 × 5 + 20) = 7 × 7 × 30. Compared to other methods employing sliding window and region proposal techniques, the YOLO model encodes more contextual information as it reasons the entire image during training and testing. Although YOLO is fast, it produces more localization errors and lower recall compared to other contemporary region-based methods (e.g., networks in the R-CNN families). On the other hand, as a 7 × 7 grid pattern is used in Redmon et al. [36], and only one object can be detected per grid, the maximum number of classes YOLO can detect is limited to 49. Moreover, if multiple objects are assigned to one grid cell, the YOLO model cannot detect all of them.

To further improve the YOLO model, Redmon and Farhadi [37] developed the YOLOv2 model by introducing batch normalization in all the Conv layers, and high resolution classifier, leading to an increase of 2 and 4% mean Average Precision, respectively. The new YOLOv2 model also introduced a new backbone classification model to maintain a balance between the network’s complexity and accuracy. In addition, to address the issue of single-object detection per grid as observed in YOLO architecture, YOLOv2 employs the idea of anchor boxes which enables multi-object detection per grid cell. Selection of anchor box dimensions can be aided by K-means clustering to search for the trade-off between the model complexity and the number of anchors to attain desired accuracy. These anchor boxes will be used as the basis to predict the final bounding box locations. The general architecture of YOLOv2 is similar to that of YOLO, except the aforementioned newly added features. The final output tensor size is S × S × B × (5 + C), which indicates each grid cell has B boxes and each box has 4 position parameters and 1 confidence score associated with it. It has been shown that YOLOv2 greatly improve the recall and localization precision over YOLO, while achieving the speed 10 times faster than Faster R-CNN on the VOC 2007 database. Further, inspired by other related works, YOLOv3 [38] was developed with several changes described as follows. First, there is a change in loss function for class prediction. Specifically, the logistic classifier is employed in YOLOv3, rather than softmax as in many other models. The use of independent logistic classifiers allows an object to be detected as a “cat” class and as an “animal” class at the same time. On the other hand, as the YOLO and YOLOv2 models struggle in detecting small objects, YOLOv3 introduced shortcut connections (similar to the concept in ResNet), which allows more semantic information to be used through combining the finer details in feature maps produced at earlier layers with the unsampled features. Lastly, Unlike YOLO and YOLOv2, YOLOv3 generate bounding boxes at three different scales where 3 boxes are generated at each scale for all grid cells, leading to the output size of S × S × B × (5 + C) at each scale. Later, YOLOv4 was developed by Bochkovskiy et al. [39]. While the main architecture of YOLOv4 stays similar to YOLOv3, it adopts multiple existing techniques that are known to improve CNN performance, such as Weighted-Residual-Connections (WRC), Cross Stage Partial connections (CSP), Cross mini-Batch Normalization (CmBN), Self-adversarial training (SAT), Mish activation, Mosaic data augmentation, DropBlock regularization, CIoU loss. To limit the scope, this review paper does not provide a detailed discussion of these techniques. Readers are referred to the respective paper or online resources to gain knowledge and implement these techniques. In summary, it has been shown that YOLOv4 improves the mAP by 10% and FPS by 12% from YOLOv3.

Sooner after YOLOv4 was published, YOLOv5 was released by Jocher [40]. The main attraction of YOLOv5 is that it came naively in PyTorch, with numerous support from Ultralytics official Github page. As it is implemented in PyTorch, it allows users to easily customize internal architecture and deploy an end-to-end pipeline at ease. YOLOv5 is similar to YOLOv4, except that it includes novel Mosaic data augmentation technique and auto-learning anchors. Further, YOLOv6 [41] was released with the updated backbone (which employs RepConv), neck, and detection head. The concept of RepConv was proposed by Ding et al. (2021) which combines 3 × 3 convolution, 1 × 1 convolution, and identity connection in one single layer. The YOLOv6 model also uses task alignment learning (TAL) to replace the IoU metric for class assignment, new loss functions, and leverages common tricks such as self-distillation and larger training epochs to further boost the performance. Later, YOLOv7 was proposed [42]. It has several innovations including the extended efficient layer aggregation networks (E-ELAN) which can improve the use of parameters and feature learning at different feature maps, a novel model scaling strategy that scales the model depth and width in concert, a modified RepConv (without identity connection), and a new label assignment method that guides both lead and auxiliary heads to generate the so-called coarse-to-fine hierarchical labels. Lastly, YOLOv8 [43], the latest version in the YOLO family, was proposed by Jocher (2023) which didn’t come with a research paper. This SOTA model employs a modified version of CSPDarknet53 (Redmon and Farhadi 2018) as the backbone, adopts an anchor-free approach, and predicts the center of objects directly, thus reducing the post-processing time by non-maximal suppression (NMS) used in many object detection models. YOLOv8 has demonstrated its excellent performance in COCO and Roboflow 100. In summary, benefiting from the official support from Ultralytics and the large YOLO community, the SOTA YOLO models provide multiple features to users including highly customizable architecture, rich sources of pretrained models, high precision, fast real-time speed, adaptive training, and advanced data augmentation, etc.

#### Pixel-wise segmentation models

##### Fully Convolution Networks

In general, CNN-based semantic segmentation models contain the encoder and decoder modules (Fig. 4). The encoder network is typically based on a pretrained classification network as the backbone, which downsamples input images, aggregates features, and extracts higher semantic information at multiple levels. The decoder is an upsampling network incorporating deconvolution (transpose convolution) layers to decode the output of the encoder to recover the spatial information. For a given image, the final output is to generate dense pixel-wise classification (i.e., to predict an image mask where every pixel of the input image is assigned a class label). Following the network architecture mentioned, the Fully Convolution Networks (FCN), proposed by Long et al. [44], is one of the most fundamental CNN models for image segmentation tasks, which initially employed AlexNet, VGG, and GoogleNet, respectively, as the backbone encoder. It had shown excellent performance in the PASCAL VOC 2011 tests compared to former segmentation models. Section 3.2.2 provides the theoretical aspects of the prevalent segmentation models in the area of SHM, while their applications for SDD research are reviewed in Sect. 4.

##### Mask R-CNN

Mask R-CNN [45] is built upon Faster R-CNN. Compared to the Faster R-CNN model, the main contribution of Mask R-CNN is supplementing an additional network overhead to perform instance segmentation (i.e., prediction of pixel-wise object masks). Also, Mask R-CNN introduced two critical features including ROIAlign and Feature Pyramid Network (FPN). To calculate the value at sampling points in ROI bins, the ROIAlign module uses bilinear interpolation from neighboring gird points on the feature map. This feature greatly addresses the misalignment issues between the ROI and the extracted features as observed in the traditional ROIPool module, leading to higher segmentation accuracy, particularly for relatively small objects. The FPN backbone constructs a multi-scale feature pyramid inside the model, thus providing Mask R-CNN with a more comprehensive network scheme to deal with multi-size objects. The additional overhead utilizes the output from ROIAlign to generate pixel-wise masks for objects. This head generally consists of several Conv blocks followed by deconvolution blocks for upsampling. The main advantages of Mask R-CNN include (1) It is relatively easy to train and deploy because it only adds a small overhead to the Faster R-CNN model for segmentation. (2) At the time of release, it outperformed contemporary, single-model entries on the related image processing tasks. (3) It runs reasonably fast, offering bounding box prediction, instance segmentation, and key point detection concurrently at 5 FPS on a single Nvidia Tesla M40 GPU. As a result, many SDD research employs Mask R-CNN, as will be seen in Sect. 4.

##### U-net

U-net is a semantic segmentation model, originally proposed for biomedical image segmentation [46]. It was built upon the FCN architecture, including the encoder and decoder networks. The encoder network is typically based on a pretrained classification network as the backbone, which downsamples input images into feature maps at multiple levels. The decoder is an upsampling network incorporating deconvolution (transpose convolution) layers and the concatenation with the higher resolution feature maps. This is one of the main contributions of U-net, since such design facilitates better learning of representation in the upsampling process using the prior high-resolution information in the encoder network.

##### DeepLab

DeepLab is another popular model in SDD research. The first version of DeepLab (DeepLabv1) was introduced by Chen et al. [46] which proposed a novel architecture combining the fully-connected Conditional Random Fields (CRFs) with CNNs. The authors introduced a new type of convolution, named atrous (or dilated) convolution, which can be used to enlarge the field of view of filters without changing the number of computational parameters. The field of view can be adjusted using the atrous rate. The further enhance the segmentation outcome by the CNN, the fully-connected CRFs are adopted to iteratively smoothen the segmentation boundaries. Later, DeepLabv2 [47] was proposed, which has a similar architecture to DeepLabv1, except that DeepLabv2 introduced the Atous Spatial Pyramid Pooling (ASPP). The ASPP is an atrous variant of the SPP which originated from the SPPNet. The main objective of the ASPP is to deal with different object scales to enhance the overall accuracy. However, as the CRF is a post-processing method employed in both DeepLabv1 and DeepLabv2, these two models cannot achieve an end-to-end and real-time prediction. Such an issue was addressed by Deeplabv3 [48], which enhanced the ASPP module with batch normalization and image-level features, and removed the CRFs to allow for end-to-end learning. Further, Deeplabv3+ was developed [49]. Figure shows a schematic of Deeplabv3+ architecture, using the encoder–decoder architecture with additional novel features. Compared to Deeplabv3, one of the key features of the Deeplabv3+ is the adaptation of depth-wise separable convolution to the atrous convolution in Deeplabv3. The depth-wise separable convolution allows the operation to be done for each input channel separately, which can greatly reduce the computational time compared to the standard convolution [50]. The combination of the depth-wise separable convolution and the atrous convolution can greatly lower the computational cost, particularly in processing high-resolution images. In addition, Deeplabv3+ also introduced a simple yet effective decoder module. It first applies a bilinear 4× upsampling to the encoder features and concatenates with low-level features that have a reduced number of channels by 1×1 convolution. Further, 3×3 convolution is applied which is followed by a 4× upsampling. This process has been shown to surpass a simple one bilinear 16 × upsampling, which can greatly help refining object boundaries.

##### Attention mechanism and transformer-based models

Recent years have witnessed the success of CNN methods in CV-based structural damage evaluation through numerous laboratory tests and field demonstrations. On the other hand, the new architecture, transformer, (originally proposed in the natural language processing field), has started gaining attraction in the SHM field. Compared to CNN, the attention mechanisms enable a broader receptive field, which can help capture the hidden connections between inputs and cross-tasks [51]. Transformer architecture is very popular and has proven to outperform deep learning architectures in natural language processing (NLP) tasks. Based on the attention mechanisms, Vision Transformer or ViT [52] and Swin Transformer [53] have been proposed for image processing tasks. The development of Transformer architecture in the CV field only started recently, and their deployment in CV-based SDD research remains highly limited. To date, there are several model variants based on the ViT architecture. In general, the ViT architecture includes the following key components: image patch generation and flattening, patch and provisional embeddings, Transformer encoder, and Multi-Layer Perceptrons (MLP) head. The transformer block includes a multi-head self-attention layer which calculates attention weights for each image pixels with the surrounding pixels, and a feed-forward layer which applies nonlinear mapping. The use of multi-head attention enables the model to attend different patches of the input sequence concurrently. The patch and positional embedding block can help divide an image into patches and map them to high dimensional vectors. These embeddings are input into the Transformer encoder. Finally, in an image classification task, the MLP head which is typically a fully connected layer, is added for class prediction.

Traditional transformer architecture processes images patch by patch, which is relatively inefficient. Swin Transformer, published by Microsoft researchers, is more efficient and accurate. To achieve this, Swin Transformer first divides images into non-overlapping shifted windows which prevents patch overlapping and at the same time allows cross-window connections. This is one of the key innovations of Swin Transformer. Another innovation is the hierarchical design of Swin Transformer, which alleviates the quadratic model complexity as seen in many former transformer models, and offers flexibility in processing images at different resolutions. As a result, Swin Transformer can be used as an effective backbone model for various CV tasks such as image classification, object detection, and semantic segmentation. To prepare input data for the implementation of the Swin Transformer model, the Auto-Image-Processor API can be used for image preprocessing such as image augmentation and normalization.

In summary, transformer models tend to capture more global dependencies and contextual information in images, which can lead to higher performance in certain scenarios such as large datasets. However, transformer models usually have more model parameters than CNNs, making them have higher memory demand, and take more effort and time to train. This may hinder their performance and applicability when the computational resources are limited, or the datasets are relatively small as in many civil engineering tasks. The selection of CNNs and transformer models depends on the computational constraint, the size of available datasets, and the balance between model accuracy and speed.

### CV Algorithms for Damage Quantification

Structural damage quantification can be achieved by measuring the dimensions of damage in 2D or 3D space. In general, in-plane damage may be quantified in 2D space using 2D computer vision methods, while out-of-plane or three-dimensional damage can be quantified using 3D computer vision methods together with 3D point cloud processing. The pros and cons of the CV algorithms for damage quantification are summarized in Table 6.

**Table 6:** Summary of CV algorithms for damage quantification

Major CV algorithms for damage quantification	Advantages	Disadvantages
Pixel-level line detection, contour detection, skeleton extraction, distance transform, etc.	Relatively fast speed	Quantification of damage is limited to 2D space
3D reconstruction and 3D point cloud processing	Accurate measurements in 3D space	Can be computationally expensive for high-quality 3D reconstruction and dense point cloud processing

#### Quantification by image pixels

Structural damage can be quantified through the processing of 2D images to measure the damaged regions in the pixel space which may be converted to real-world dimensions using a pre-calibrated setting. The general process is relatively straightforward. First, the damage region is localized by vision algorithms using either bounding boxes or pixel-wise masks which can be generated using image binarization, CNNs, or attention-based models, etc. Second, these predicted boxes or pixel masks are further processed using methods like morphological operation to extract damage metrics, such as concrete crack width, concrete spalling dimensions, steel rust (corrosion) ratio, steel bolt loosening, etc. Recent applications of these quantification methods are reviewed in Sect. 4.

These methods are categorized as 2D vision methods, which can only provide in-plane measurements, where the target structural surface is generally required to be flat or nearly flat. Otherwise, the quantification accuracy will be compromised. The main limitation of these 2D vision methods is that they are unable to evaluate out-of-plane metrics (e.g., concrete spalling depth, out-of-plane deformation of steel plates).

#### Quantification by 3D Point Clouds

**Fig. 5:**
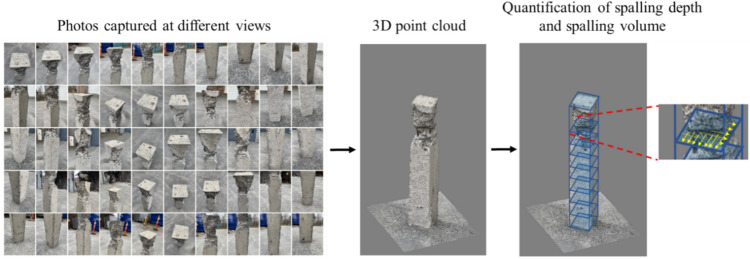
3D point cloud-based concrete spalling quantification

Having realized the limitations of 2D vision-based damage quantification methods, over the past few years, there has been a growing trend to adopt 3D vision techniques to quantify structural damage. In this case, Structure-from-motion (SfM) algorithms can be adopted, which take photos captured from multiple views as the input and reconstruct a 3D dense point cloud or 3D object surface model. The reconstructed 3D model will be further processed to quantify structural damage in 3D space. Figure 5 depicts an example [54] of using vision-reconstructed 3D point clouds to quantify concrete spalling volume. Recent applications of 3D model-based damage quantification methods are provided in Sect. 4.

## Applications of CV Algorithms for SDD

Table 7 presents a summary of recent SDD research with the associated CV methods, including the system-level evaluation, and component-level evaluation classified into different damage types under four structural categories including concrete, masonry, steel, and timber structures. Representative studies including their research highlights and limitations are discussed hereafter.

**Table 7:** Recent CV-based SDD research: algorithms and applications

Topics of damage evaluation	Methodology and application examples
*System-level*	
	Classification: Machine learning models: Support Vector Machine, Random Forest, Decision Trees, etc [55] CNNs: AlexNet, GoogleNet, VGG, ResNet, MobileNet, etc. [28, 56–59] Attention-based mechanisms/Transformer: Swin Transformer [60] Localization: CNNs: Mask R-CNN [61], YOLO [62] 3D point cloud processing [63]
*Component level*	
Concrete structures	
Concrete cracks	Classification: Machine learning models: Support Vector Machine, Random Forest, Decision Trees, Generative Adversarial Network, etc. [64–68] CNNs: AlexNet, GoogleNet, VGG, ResNet, Inception, and Xception, etc. [28, 69–80]; customized models [81–84] Attention-based mechanisms/Transformer [85] Localization: Edge detection/segmentation: Image binarization/thresholding [64, 70, 72, 76, 83, 84, 86–89] active contour model [73] Machine learning models: Support Vector Machine, Random Forest, Decision Trees, Generative Adversarial Network, etc. [90] CNNs: Faster R-CNN [91–94], Mask R-CNN [95, 96] YOLO [97–101] SegNet [102], U-net [78, 103–112] FCN [113, 114] DeepLab [115], customized models [81, 116–118] Attention-based mechanisms/Transformer [119–124] 3D point cloud processing [125, 126] Quantification: Morphological operation such as skeleton extraction and distance transform methods [70, 72, 73, 86, 93, 105, 123] Regression-based prediction [89, 127, 128] DIC and wavelet analysis [129, 130] 3D reconstruction/point cloud processing [87, 94, 109, 111, 131, 132]
Concrete spalling	Classification CNNs: AlexNet, GoogleNet, VGG, ResNet, Inception, and Xception [28, 56, 74, 77, 78, 133] customized models [75, 134] Localization: CNNs: Faster R-CNN [91], Mask R-CNN [135, 136] YOLO [100, 106], FCN [113, 137], U-net [78, 137, 124] DeepLab [29, 115, 137] Attention-based mechanisms/Transformer [60, 138] Quantification 3D point cloud processing [54, 132, 136]
Concrete subsurface delamination	Classification: CNNs: VGG, ResNet, Xception and MobileNet [139] Localization: K-means clustering [140, 141] CNNs: DeepLab [142] Thresholding [143–146]
Steel reinforcement damage in RC structures	Classification CNNs: AlexNet, VGG, ResNet, Xception and MobileNet [77] Localization: CNNs: Faster R-CNN [91, 94], DeepLab [115], YOLO [28, 29, 100, 106], U-net [112]
*Steel structures*	
Steel cracks	Classification: CNNs: VGG [147], customized models [148] Localization: CNNs: YOLO [149], FCN [150, 151], U-net [152] DeepLab [149, 153], deep fusion CNN [148]
Steel corrosion	Localization: CNNs: Faster R-CNN [154], PANet [149, 155] U-net [156], customized models [157]
Steel deformation, flatness and fracture	Classification: CNNs: DenseNet [158] Localization: CNNs: YOLO [159] Quantification: Contour detection [160] 3D point cloud processing [161]
Steel bolt loosening	Classification: Support vector machine [162] Localization: CNNs: Faster R-CNN [163], YOLO [164] Orientation-aware CNN [165], 3D bounding box fusion [166, 167] Rotation-based quantification: HT algorithm [168–170] Tracking algorithms: SSD-based tracker [171], KLT tracker [164] Exposed shank-based quantification: Bounding box characteristics [163, 165] 3D point cloud processing [166, 167]
*Masonry structures*	
Masonry cracks, spalling, efflorescence	Classification Machine learning models: Support Vector Machine, Random Forest, Decision Trees, Generative Adversarial Network, etc [172] CNNs: AlexNet, GoogleNet, MobileNet [173, 174] customized models [172, 175] Localization: CNNs: Faster R-CNN, Mask R-CNN, U-Net, FCN, FPN, and DeepLab [174, 176, 177]
*Timber structures*	
Timber defects (Rot damage, insect damage, knot, mildew, pinhole, etc.)	Classification: CNNs: ResNet, VGG, Inception, EfficientNet [178, 179] Localization: CNNs: SSD [180]

### System-Level Evaluation

In many cases, system-level evaluation relies on the classification of images captured for the entire structural system. The images of structures are generally classified by pretrained classification models into (1) damaged or undamaged, (2) collapse, partial collapse, and complete collapse, or (3) multiple damage states with reference to the post-disaster damage rating guidelines such as FEMA. CV-based system-level classification models have been investigated using CNNs and attention-based mechanisms or transformer architecture to analyze images captured on site. Over the years, these methods were focused on exploring different model architectures and training options to examine the algorithms’ accuracy and applicability in dealing with different scenarios (e.g., post-earthquakes, post-hurricane, etc.). It has been shown with proper training settings, most of these methods can achieve highly satisfactory accuracy (i.e., close to 100%). However, many of the studies utilized small datasets for training and validation, meaning the generalizability of these pretrained models to a different scenario needs further investigation. On the other hand, studies using infrared images for system-level evaluation have also been attempted. For example, Kargin et al. [62] employed a YOLOv3 algorithm to detect buildings from satellite infrared imagery. By analyzing heat leakage changes over time, the building damage was classified into low, minor, moderate, and high. The proposed method can be used as a long-term monitoring solution to capture the progressive growth of damage. However, their method struggles to detect buildings with minor damage, and is also affected by climate effects.

### Component-Level Evaluation

Most recent studies have been focused on investigating CV methods for component-level damage evaluation. This section aims to provide a detailed review of related case studies across multiple structural categories including concrete, steel, masonry, and timber, at three hierarchies (i.e., damage recognition, localization, and quantification). The contributions and limitations of these studies are discussed.

#### Concrete Structures

Concrete is one of the most widely used construction materials for civil infrastructure worldwide. Detection of concrete damage is important to maintain the functionalities of concrete infrastructure. This section reviews common critical concrete damage types (concerning the structural performance) addressed by the recent CV-based SDD research.

##### Concrete Cracks

Concrete cracks are the most common damage type of concrete structures. In fact, most of the recent CV-based SDD research for concrete structures is focused on crack assessment, which has made remarkable progress in enhancing crack recognition, localization, and quantification accuracy, using different methods such as the machine learning models (e.g., Support Vector Machine, Random Forest, Decision Trees, Generative Adversarial Network, etc.), CNNs, attention-based architecture, and vision-based 3D reconstruction together with 3D point cloud processing. The below paragraphs provide a detailed review of recent advances of representative applications of CV-based concrete crack evaluation. Research about crack detection and localization is first reviewed, followed by crack quantification research.

Concrete crack classification models have been investigated. For example, Gharehbaghi et al. [74] developed FastCrackNet to identify concrete cracks under noisy and shadow conditions. By integrating wavelet image processing, LSDA feature conditioning, and a 12-layer deep network, the system adeptly handled challenges like motion blur and salt and pepper noise. When benchmarked against renowned CNN models such as Xception and GoogleNet, FastCrackNet demonstrated competitive performance in terms of accuracy and speed, showing resilience to various environmental challenges. The concept may be further investigated when detecting other types of damage in noisy images captured under adverse effects.

Localization of concrete cracks can be achieved using one-stage or two-stage methods. Earlier research generally employs two-stage methods, which typically involve the identification of crack regions using the sliding window technique, followed by crack classification, or segmentation using image binarization and thresholding. For example, Kim et al. [81] proposed a two-stage crack detection method, which first identifies the so-called concept of crack candidate region (CCR) from the original images, followed by a classifier like CNN-based model to classify the CCR into cracks and non-cracks. As a result, the CCR classified as cracks can be combined with image binarization for crack localization. Although the results were promising, the adopted image binarization method is relatively sensitive to the selected threshold, and may not perform well in processing low-contrast images with high level of noise. On the other hand, the procedures are relatively time-consuming. Later, one-stage methods (i.e., CNN with the encoder-decoder architecture) gained more attraction as they can directly output pixel-level prediction (masks) for crack patterns in a single pipeline, which enables end-to-end training and deployment, thus being more efficient and convenient to use. For example, Li et al. [96] employed the Mask R-CNN method for concrete crack segmentation. The study also explored an image enhancement method to refine and delineate concrete cracks in images with degraded quality during data collection. The research emphasized the integration of two advanced deep neural networks to address the common issue of image degradation due to environmental factors. The study showed that with image enhancement, their proposed method could significantly improve image clarity, especially in challenging conditions. The results highlighted an 8-13% boost in prediction accuracy, suggesting the potential of combining GAN and the image enhancement method in a unified model for future studies to further optimize crack identification processes. Another very popular one-stage method for crack segmentation is U-net. For example, Zhang et al. [103] proposed CrackUnet with four different depths, based on the U-net architecture to detect concrete surface cracks at pixel level. The research applied a new loss function to address the training problem posed by sample imbalance. The study examined how the size of the dataset and the depth of the model influenced the performance of the DL-based model and showcased its generalization on a public dataset. It was shown that larger datasets bolstered the model's detection performance, with the depth of the model not linearly increasing its effectiveness. Kang et al. [93] employed U-net for detecting concrete cracks in images. By using the Monte Carlo dropout (MCDO) strategy, the framework identifies images with high uncertainty to optimize model training. In was shown that with only 25% of the training data from an open-source dataset, the model achieved 99.2% of the performance of a fully-trained counterpart, showcasing the efficiency of this approach in concrete crack detection. However, the uncertainty-based method in their study was focused on epistemic uncertainty arising from the insufficient amount of training data. It was suggested aleatoric uncertainty should be formulated in future studies. In addition, the MCDO strategy can also be potentially extended to training of other defect types. More recently, research using attention-based mechanisms for crack evaluation has been attempted. For instance, Hang et al. [124] introduced an attention-based feature fusion network for concrete crack detection. The research emphasized the integration of two attention mechanisms, the vertical and horizontal compression attention module and the efficient channel attention upsampling module can enhance segmentation accuracy. Experiments conducted on various datasets revealed that the model can achieve a promising IoU with finetuning. It was summarized further studies may focus on exploring the relationship between the identified optimal learning rate and the attention mechanisms to further elevate the model's performance in challenging environments. In summary, concrete crack detection and localization algorithms have received the most attention among all the damage types across all the structural categories. Many of the existing crack evaluation methods were developed based on CNN architecture with various training options and performance boosting strategies, while more recently, research started to adopt attention-based mechanisms.

Apart from the concrete crack detection and localization methods, crack quantification methods have also been proposed as reviewed below. Cracks can be quantified by applying the morphological operation to predicted masking regions which are generated by one-stage or two-stage methods aforementioned. For example, Kim et al. [86] proposed a concrete crack quantification method. The study first segmented concrete cracks, where the segmented cracks were quantified by patterns, width, and length, following three steps including thinning of crack area, tracking of crack spline line, and orthogonal profiling of cracks. Although the quantification steps were inspiring, the inspection pipeline incorporated an image binarization and thresholding method, Otsu’s algorithm [104], which is sensitive to the selected threshold, and can only deal with localized image patches where the edge features of the images are relatively simple. Further studies can be explored by combining the proposed morphological methods with one-stage crack localization models (e.g., U-net), which may lead to high accuracy and robustness. Some other popular crack quantification methods rely on the use of 3D point clouds. Kim et al. [131] employed a stereo camera system to detect and quantify concrete cracks in 3D space. The stereo camera is capable of generating 3D point clouds where the predicted 2D bounding boxes can be projected into 3D space. Further, the crack width and 3D locations can be estimated accordingly. Field validation on an in-service railroad bridge demonstrated the effectiveness of the proposed system. It was suggested future directions include UAV implementation, accounting for more types of surface damage, and refining depth estimation for curved surfaces. On the other hand, regression-based methods were also explored for predicting crack-related damage quantities. For example, Laxman et al. [128] proposed a crack detection and crack depth prediction model that combines a customized CNN with regression models such as random forest and XGBoost. Specifically, the CNN performs the binary classification of images into crack and non-cracks. For the crack images, the CNN-extracted features and labels are fed into the regression models to predict the crack depth. Despite the promising results, the method was only validated on a reinforced concrete slab in the laboratory. Further investigation of the generalizability of the regression models to other concrete components may be conducted to establish a potential standard for model adaptation.

In general, despite the achievements, the CV methods investigated in these studies cannot distinguish structural and nonstructural cracks. Also, almost no attempts have been made to associate the crack patterns with specific failure modes (e.g., shear cracks). Due to the load path and load-carrying mechanisms in reinforced concrete, structural cracks are typically formed in specific regions with expected orientations, as a single continuous crack or multiple parallel cracks. For example, the steel reinforcement corrosion-induced cracking on an RC column is typically developed in the longitudinal direction of the reinforcement. The nonstructural cracks have more inconsistent patterns, which are caused by many different factors such as concrete own properties and environmental effects like temperature and humidity. Future research can be explored to distinguish cracks due to different causes and failure modes, thus providing more useful information to understand the structural behavior and guide maintenance decisions thereafter.

##### Concrete Spalling

Concrete spalling is another major damage type of concrete structures, which involves a noticeable amount of materials spalled off from the concrete surface. Due to the complex shapes of spalling, it is generally localized using image segmentation methods. Similar to concrete crack evaluation, there exist one-stage and two-stage methods for concrete crack recognition and localization. Chow et al. [133] employed a two-stage method that combines a customized CNN, ResNet, and sliding window technique to detect concrete spalling and cracks. The method followed three consecutive steps including defect recognition, defect extraction, and defect classification. The inspection scheme is relatively time-consuming as it employs the inefficient sliding window technique and passes through two different CNNs in series. Later, Kim and Cho [135] employed a one-stage model, Mask R-CNN, which was trained on 765 concrete images, capturing various damages such as spalling, cracks, efflorescence, and rebar exposure. The validation was performed on 25 actual test images, yielding an average precision of 90.41% and recall of 90.81% for localization and 87.24% precision and 87.58% recall for segmentation. The study showed the effectiveness of Mask R-CNN in segmenting multiple concrete damage types, but it was only validated on very small datasets. Li and Zhao [137] proposed a performance improvement strategy for concrete damage detection using stacking ensemble learning of multiple one-stage semantic segmentation models. The study investigated multiple CNN-based methods such as FCN, U-Net, and DeepLabv3+ to detect concrete spalling and cracks. With the ensemble learning strategy, it was shown their proposed methodology can effectively enhance the detection performance of a single semantic segmentation network in various metrics such as mean IoU, and FWIoU. Despite the promising results, the study was only validated on a small dataset. Further investigation of the ensemble methods and more damage types should be carried out. More recently, research employing the attention mechanism for spalling evaluation has been attempted. For example, Kim et al. [138] proposed an integrated method by incorporating a max-mean pooling module and attention-base node into the CNN-based classification backbone, to recognize concrete spalling, delamination, and cracks, achieving a defect detection accuracy of over 95%. The authors suggested that further investigation is needed for the proposed method to incorporate more damage types and be validated under changing lighting conditions and more complex concrete surfaces. Gao et al. [60] have proposed a novel transformer-based architecture to evaluate concrete spalling. Their proposed framework allows for multiple attributes at different levels to be considered in a single damage detection pipeline, ranging from the scene level, structural system level, structural component level, and local damage level. The framework also performs end-to-end multi-tasking including image classification (e.g., damage state, failure modes), object detection, and semantic segmentation (e.g., localization of concrete spalls). It has been concluded that transformer-based architecture not only achieves comparative accuracy to the CNN-based architecture, but can also be easily integrated with multi-task and transfer learning to facilitate a more convenient training and deployment process.

Apart from the detection and localization of concrete spalling, quantification methods of spalling have also been explored, which involve the estimation of multiple damage metrics such as the largest spalling dimension, spalling depth, and spalling volume. For example, Yuan et al. [132] proposed a 3D vision-based method to localize and quantify concrete cracks and spalling. By leveraging the advantages of the Internet of Things (IoT) for data communication, the proposed system uses a stereo camera for 3D vision-based reconstruction of structural components. Then, Mask R-CNN was adopted to segment concrete cracks and spalling, where the segmentation results were projected to the 3D point clouds to quantify concrete crack dimensions and spalling volume. The proposed method was implemented on a novel intelligent inspection robot equipped with stereo vision for the detection and quantification of spalling in 3D space (e.g., spalling depth or volume). In their experimental validation, the proposed system was shown to effectively segment, localize, and quantify the damage of a reinforced concrete column with notable accuracy. This study highlighted the inefficiencies of existing 2D vision-based segmentation methods in capturing the true size of concrete damage. Despite the promising outcomes, their proposed method was only validated on a simple concrete component, which should be further extended to other types of concrete structures with more complex geometries.

##### Concrete Subsurface Delamination

Concrete subsurface delamination can occur due to many different factors such as corrosion of steel reinforcement and improper curing. Delamination can further evolve into large cracks and spalling, which can greatly reduce the structural stiffness and load-carrying capacity. Therefore, it is important to earlier identify delamination and apply an appropriate retrofit scheme to maintain the structural performance. Traditionally, delamination may be manually inspected using hammers. However, such an approach may further impact the delaminated portion. In order to achieve non-destructive inspection, researchers have investigated CV-based methods to detect subsurface delamination. As subsurface damage cannot be captured by RGB cameras, Infrared Thermography (IRT) cameras can be considered. The IRT cameras can generate thermal images that capture the temperature difference between the delaminated portion and the normal portion. These thermal images can be further processed by CV methods to evaluate the delamination.

Earlier applications of IRT methods mainly employ traditional image processing techniques such as binarization, thresholding, gradient analysis, and bob analysis, etc. [143–145]. More recently, machine learning and data-driven methods have been established to analyze thermal images. For example, Omar et al. (2018) employed k-means clustering as a value-based segmentation method. With a predefined threshold, the method can cluster the thermogram of the concrete surface into normal, delaminated, and monitoring-required regions. Meanwhile, Omar and Nehdi [140] implemented a similar approach with drones to achieve remote inspection. McLaughlin et al. [142] employed the DeepLab segmentation model with the MobileNet as the backbone to segment concrete delamination and spalling and implemented with a ground robot. Pozzer et al. [139] investigated several CNN models (VGG, ResNet, Xception, and MobileNet) to localize delamination, cracks, spalling from both thermal and optical images. These studies were validated on full-scale concrete bridge components. Despite the promising outcomes, the CNNs used in these studies were trained and validated by small datasets. Future research should be explored in examining the CNN methods for thermal images of various structural concrete components under various environmental conditions (e.g., different temperatures and lighting conditions). A recent study by Ichi and Dorafshan [146] employed an adaptive thresholding method for accurate delamination detection. By extensively investigating the effects of threshold values, delamination depth, and ambient environmental factors on the model performance, the authors devised an optimized detection model, which was found to outperform the k-means clustering method proposed by Omar et al. [141].

In summary, the performance of the IRT image processing is affected by the concrete cover thickness and depth of delamination. Also, as the methods rely on temperature gradient, the performance is also affected by the weather and climate.

#### Steel Structures

Recent applications of CV methods in the damage detection of steel structures are pertaining to steel surface cracks, steel corrosion, deformation measurement and fracture detection, and steel bolt loosening.

##### Steel Surface Cracks

Steel surface cracks can be caused by fatigue, minor scratches, welding, or grinding processes. In general, CV-based steel crack detection methods are similar to those for concrete crack detection methods, as reviewed below.

Earlier research was more focused on qualitative assessment. For example, Dung et al. [147] employed CNN-based methods to classify steel crack images of gusset plate joints. The study only classifies images into “crack” and “non-crack”, without further distinguishing the crack types such as fatigue cracks or minor scratches. Later, steel crack localization methods were explored, including multi-stage methods and single-stage methods. For example, Han et al. [149] proposed a three-stage crack detection method which includes a filter based on simple linear iterative clustering (SLIC) to segment original images, YOLOv3 to detect smaller cracks, and finally DeepLabv3+ model for image segmentation. The authors concluded that by considering the intersection of detection outcomes from these three methods, the overall crack localization capability and accuracy can be enhanced. Quqa et al. [148] proposed a steel fatigue crack detection method for steel bridge components. The original image is first divided into subregions which is then processed by a CNN-based classification model as “damaged” and “undamaged”. Secondly, morphological operations are implemented to reduce incorrect classification by fixing the discontinuities in the gap regions between damaged subregions. The third step involves the use of Sobel edge detection method to detect cracks on the subregions which are labeled as “damaged”. The main limitation is the Sobel edge detection method is relatively sensitive to noise. On the other hand, minor cracks may be distributed over a structural surface. The assumption of cracks being continuous (as in the second step) may wrongly connect these minor cracks into a major crack, leading to false detection. Dong et al. [152] employed a single-stage detector, U-net, to segment steel fatigue cracks where the U-net model is applied to small image patches partitioned from the original large images collected on site, and the detection result is concatenated afterwards. It is shown that during the inspection of fatigue cracks, annotations and rulers may be used and presented in the collected images, leading to a more complex image background. As the textural features of background objects (e.g., edges or shadows of the ruler) may be similar to those of fatigue cracks, the model trained in small dataset may wrongly recognize other background objects as cracks, and consequently may not be generalized well at a broader scale. Zhu et al. [150] proposed the CrackDet, using the FCN-based architecture to identify steel surface cracks from steel girder images. The study attempted to investigate various loss functions and additional feature extraction methods (i.e., dilated convolution, and spatial pyramid pooling) to enhance the network’s capability to segment fine cracks in a relatively complicated background. It was suggested that the IoU-based loss functions lead to higher performance, and may be used in conjunction with the proposed CrackDet in a two-step scheme in future studies to further enhance the segmentation accuracy. In a similar study, Ta et al. [153] employed the DeepLabv3+ network which incorporates atrous convolution and spatial pyramid pooling to identify steel cracks. The study explored the effects of background objects (e.g., handwriting markers, and weld lines) on the detection performance. Specifically, three segmentation scenarios were considered, including two classes (cracks and background), four classes (crack, ruler, mark, and background), and five different classes (crack, ruler, mark, contour and background). It was suggested that the two-class segmentation case yields higher performance, and further enhancement may be achieved using the class-pixel label balancing approach and adaptive image-cutting techniques. Xu et al. [181] employed a deep fusion convolutional neural network (FCNN) to segment fatigue cracks and handwritten markers on the steel box girder surface from the background. It has been shown the use of multiple-level and multi-scale image features makes the FCNN superior to the regular CNN in crack identification. In addition, the authors also investigated the effects of the image super-resolution process on the model performance and found that such a process will have a negative impact possibly due to the crack percentage in an image being small.

Despite the achievements, existing studies did not make efforts to distinguish minor scratches and fatigue cracks. While the former is less critical, the latter is of much more concern with respect to structural performance and integrity. This remains one of the major challenges in vision-based steel crack detection.

##### Steel Corrosion

Steel corrosion is another critical damage type. The American Society for Testing and Materials (ASTM) proposes a 0-10 scale to rate the corrosion performance and provides maintenance suggestions. The corrosion percentage is a critical damage metric for structural owners and engineers for decision-making during the inspection and repair processes [182]. In CV-based SDD research, steel corrosion can be evaluated using bounding box localization or pixel-level segmentation. Earlier related studies in this subdomain can be referred to Dong and Catbas [7]. Recent related studies are discussed as follows. Luo et al. [154] employed the Faster R-CNN model to localize multiple corrosion types from steel surfaces including the nubby corrosion, faster corrosion, and bar corrosion from panoramic images of steel structures and equipment on a construction site. It was concluded that the trained Faster R-CNN is robust and effective in dealing with irregular topology and camera distortion. Han et al. [155] proposed a corrosion detection method which pre-segments images using SLIC method, followed by finer segmentation using feature pyramid network (FPN) and path aggregation network (PANet) algorithms. It was concluded that by combining these algorithms, the proposed method can reliably localize steel corrosion on large-scale structures in complex backgrounds. In addition, with geo-reference UAV captured images, the orientation and global location of the steel corrosion relative to the whole full-scale structures can be estimated. Khayatazad et al. [157] customized several simple feed-forward artificial neural networks (ANNs) with various number of hidden layers to segment steel corrosion from images with different illumination criteria and background objects. It was concluded that all the ANNs were successfully trained to achieve satisfactory performance, while the deeper ANNs generally offer higher accuracy than the ANN with a single hidden layer. Jiang et al. [156] employed a modified U-net model by adding fusion and attention module to detect multiple corrosion-related damages including corrosion, mildew and ponding. Further, a convenient equation was derived to convert detected damage pixels to real-world damage area. The method was validated on images of steel box girders collected from routine bridge inspection. It was concluded that while the modified U-net can achieve satisfactory accuracy metrics, the speed is relatively slow due to the complex model architecture. In addition, although the accuracy is promising, the dataset used in the study is very small. Further investigation is required to evaluate the feasibility of the proposed model for other steel components. In summary, the models for steel corrosion evaluation employed in recent studies remain similar to those reviewed in Dong and Catbas [7].

##### Steel Deformations, Flatness, and Fracture

While many of the prior studies were focused on steel cracks and corrosion, it should be noted that the flatness and straightness of steel components are critical metrics to inspect according to existing inspection guidelines as will be reviewed in Sect. 5. Steel beams and steel truss members under service loads experience longitudinal axial deformation and lateral deformation due to shear and bending moment. Thin-walled steel plates are prone to buckle which results in relatively large in-plane and out-of-plane deformations. In-plane and out-of-plane deformations will greatly affect structural stiffness and capacities when exceeding the yielding and critical buckling points. Therefore, evaluating these deformation-related damage is of great importance. A recent study using CV-based deformation measurement of steel structures has been carried out [161]. In their study, SfM techniques were adopted to reconstruct 3D point clouds of steel plated structures using images captured from various viewpoints. The 3D point clouds were processed by a 3D object detection method and 3D point cloud clustering method. The research shows the proposed method can yield accurate measurement in both the in-plane and out-of-plane directions, at a much lower cost compared to the use of terrestrial laser scanners. The downside is that the SfM methods adopted in their study are relatively time-consuming, which can take up to 20 min for a full-scale steel plate wall based on experimented hardware configuration, and will accumulate further for larger steel structures. However, with the rapid advancement of computational hardware, such limitations can be greatly alleviated over time. In addition, significant deformations may cause steel surfaces to fracture. A fractured surface can greatly affect the structural stiffness and capacities, and completely change the original deformation growth patterns. A recent study [160] employed CV methods to identify and quantify steel fractures. Specifically, a contour detection method was used to identify the border of an object (e.g., steel components) with the fracture regions. To quantify the fracture region more accurately, a geometric perspective transform method was applied to align the steel surface where the fracture appears. The authors have validated the CV methods for a damaged steel beam from a real steel bridge, where the quantification outcomes were used to update the finite element model. Despite the promising outcomes, there are a few limitations noticed. There exist several manual processing steps, such as segmentation of the steel components from the original image, and selection of feature points to perform perspective transform, etc. On the other hand, the methods were only validated for simple steel structures. For steel structures with more complex geometric shapes, the contour detection method adopted may not be able to fully capture the object border and fracture regions. Future research should be conducted to address these challenges.

##### Steel Bolt Loosening

**Fig. 6:**
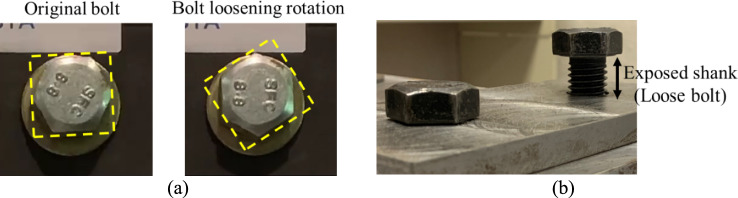
Bolt loosening evaluation by: **a** bolt rotation and **b** exposed shank.

The AASHTO inspection manual considers bolt loosening as a critical inspection requirement. In existing CV-based SDD research, bolt loosening is evaluated by either recognizing and quantifying bolt rotation, or exposed shank, as shown in Fig. 6. To capture the exposed shank, images are taken from the side or oblique view. The rotation during the loosening process is generally realized with the camera view set parallel to the bolt head or nuts, or with postprocessing using perspective transformation if the view is not strictly parallel. This section reviews the recent literature on bolt loosening evaluation based on bolt rotation and exposed shank.

Rotation-based methods generally comprise localization of bolts using image-based object detection algorithms, and quantification of bolt rotation angle based on local image features. Earlier, image registration methods and Hough Transform (HT) algorithm [183] were adopted to identify the edges of bolts and estimate the bolt rotation accordingly using two images taken at two instants before and after loosening [168, 184]. Later, the HT algorithm was further combined with CNN-based methods to achieve more robust automatic bolt localization and damage evaluation [169, 170]. However, the robustness of the HT algorithm in edge detection is not very high when images come with shadow, complex background, and high noise. Besides, the measurement range using two static images is limited to 60 degrees due to the nature of the bolt shape. To address the range limitation, Zhao et al. [171] trained an SSD algorithm to detect bolts and symbols on the bolt surface. Using object detection-based tracking, the selected symbol was continuously localized in real time by the SSD algorithm. The bolt rotation is estimated with reference to the location time history of the symbol. Although the accuracy was promising, it requires an excessive amount of training for different types of symbols appearing on different bolts, which may hamper the generalization of such a method. Further, Pan and Yang [164] proposed an automatic bolt detection and loosening evaluation method, which combines YOLOv3 and a KLT tracker [185]. In the proposed methodology, bolts are first localized by YOLOv3 which generates local Regions of Interest (ROIs) for each bolt. This eliminates the need to process a large portion of pixels in the image in the subsequent step, thus greatly reducing the computational cost. The KLT tracker is then initiated on the ROIs and continuously tracks the local feature points inside the ROIs. Finally, the bolt rotation can be extracted with reference to the motion of the feature points. Despite the real-time speed and high accuracy achieved, the method relies on local textural information (e.g., symbols, and stains on the bolt surface). Although this may not be a problem for most engineering applications, in rare situations where the bolt head surface is polished or very clean, it will require manual marking on the bolt surface to implement the method.

Other studies evaluate bolt loosening using an exposed shank of loosened bolts. The bolt loosening can be quantified using the aspect ratio of bounding boxes, or the length of the exposed shank. Earlier, a combined support vector machine (SVM) and HT method was investigated to classify bolts into loosened and non-loosened [112, 124, 148, 151, 155, 162–180, 182–186]. The method may wrongly classify lightly loosened bolts (e.g., less than 3 mm exposed shank length) as non-loosened bolts, because they are visually very similar to classification models. Later, Zhang et al. [163] trained a Faster R-CNN detector to detect tight and loosened bolts, where the bolt loosening is quantified using the exposed shank length. The quantification process requires the camera view to be strictly set perpendicular to the longitudinal axis of the exposed shank. Hence, the evaluation results are sensitive to the viewing angle of the acquired images. To relax the viewing angle constraint for bolt loosening evaluation, Zhang and Yuen [165] employed the concept of orientation-aware bounding box prediction, which can localize bolts with rotated bounding boxes and quantify bolt loosening using the aspect ratio of predicted boxes. This allows loosened bolts to be more easily recognized in images captured at an oblique view. Despite the advancement, the method also struggles to identify lightly loosened bolts, as in most 2D vision-based approaches. To enhance the capability of the bolt loosening detection using the exposed shank, Pan and Yang [167] proposed a 3D vision method where images were collected from many different views to generate a 3D point cloud of bolted connections. The 3D point cloud was then processed to extract bolts and quantify the exposed shank length in 3D space. It was shown that with accurate 3D reconstruction, their method can quantify loosened bolts, up to 1 mm looseness, which greatly enhances the ability to identify lightly loosened bolts. The main limitation is the requirement of abundant camera views for 3D reconstruction may be difficult to satisfy during the data collection process due to surrounding obstructions.

#### Masonry Structures

In general, applications of CV-based inspection of masonry structures include damage classification, localization of masonry building components or bricks, and segmentation of damage patterns. Masonry damage types investigated by recent research generally include cracks, spalling, and efflorescence. Recent advances are reviewed as follows. Hallee et al. [172] investigated different ML models (e.g., Support Vector Machine, Random Forest, CNN, etc.) for masonry damage classification, which is limited to crack damage only. Wang et al. [173] applied the concept of sliding windows on masonry images. The pretrained AlexNet and GoogleNet models were selected and finetuned to classify masonry image patches into intact, crack, efflorescence, and spall types with over 90% accuracy in all classes. On the other hand, Seo et al. [175] investigated a simple CNN model to perform classification on infrared images of masonry heritage building damage and components (e.g., cracks, windows, doors). The main limitation is the resolution of infrared images is generally very low compared to color (i.e., RGB) images, meaning that the photos need to be captured at a relatively close distance and consequently many photos are needed for a complete building damage inspection survey. On the other hand, research on masonry damage localization is also conducted. For example, Wang et al. [176] proposed a damage detection method built upon the Faster R-CNN model to localize masonry efflorescence and spalling, which can predict bounding boxes for damage patterns of various sizes, and is more superior to the fixed-size sliding windows in Wang et al. [173]. The study also attempted to implement the vision algorithm in real time with a smartphone camera to facilitate a more efficient inspection. Dais et al. [174] employed MobileNet for crack and non-crack image classification, and feature pyramid network (FPN) to segment crack patterns from images. It was concluded that the lightweight MobileNet can achieve comparative classification performance to more complex networks such as VGG, and the FPN provides similar accuracy to U-Net in crack segmentation. Dang et al. [177] employed Mask RCNN to detect bricks, and U-Net, FCN, and DeepLabV3 + to segment cracks on the brick surface. Using the prior information of brick dimensions, the crack length can be quantified in real-world dimensions with up to about 90% accuracy as reported in their study. In summary, CV-based masonry damage evaluation methods are very similar to those for concrete structures.

#### Timber Structures

Existing research about CV-based timber inspection is focused on identifying defects for quality control of wood products during the manufacturing process, rather than the structural damage of wood due to loading and environmental effects. For example, Haciefendioglu et al. [178] employed three CNN-based image classification methods to categorize wood images into dry rot damage, wet rot damage, and insect damage. Yang et al. [180] investigated the use of SSD algorithm to localize multiple damage types (e.g., live/dead knot, mildew, pinhole, etc) of wood products. A more recent study by Lee and Yu [179] employed CNN-based image classification methods (i.e., VGG, ResNet, etc) to evaluate images of wooden heritage structures. Despite the high accuracy, the study is limited to classifying local wood images into normal and abnormal status, without damage localization and quantification.

In summary, most existing studies about CV-based wood damage detection are focused on their natural defects, not structural damage caused by external factors such as loadings, aging, or natural disasters. Therefore, the existing related development of the CV algorithms in structural timber engineering remains at the infancy stage, which opens numerous opportunities for algorithm development to accurately and reliably assess structural damage, failure modes, and performance indicators of timber structures which are more useful metrics to structural engineers and researchers. For example, Palma and Steiger [187] presented a detailed review of SHM techniques of timber structures including the damage metrics of interest and the associated monitoring technologies to obtain the metrics. Without a detailed discussion, the authors envisioned that the CV techniques such as photogrammetry and digital image correlation (DIC) may be used for health monitoring of timber structures at a broader scale.

## Gap Between the Existing CV Models and Structural Inspection Guidelines

As pointed out by numerous existing studies, it is crucial to develop computer vision methods that comply with existing inspection guidelines to better support real engineering practices [7]. For example, Matos et al. [188] reviewed and compared bridge condition assessment guidelines in Italy, Slovakia, and Portugal. In Italy, the so-called Class-of-Attention determination procedures are established to quantify risk and damage at five increasing levels. In Slovakia, a load-carrying capacity coefficient affected by defects is used to assess the condition of a bridge into seven possible states. In Portugal, onsite inspection is the main approach to assess the condition of bridges, where the so-called condition state is evaluated based on damage severity, damage extension, damage development and consequences. Within these guidelines, it is noted that visual damage inspection should follow an established protocol to provide a rigorous and comprehensive damage and risk assessment of structures. Therefore, this section reviews examples of inspection guidelines (written in English) used in practice for concrete, steel, masonry, and timber structures, respectively, which sets a basis to discuss the knowledge and technology gap between the capability of existing CV models and common structural inspection guidelines.

### Existing Guidelines and Challenges: Concrete and Masonry Structures

Structural failure-related damage types of concrete and masonry are similar (e.g., crack, spalling, reinforcement damage, etc.). Therefore, this section reviews the existing inspection guidelines and challenges for concrete and masonry structures together.

Over the past decades, the American Concrete Institute (ACI) has published many documents related to concrete inspection. Other organizations such as the American Association of State Highway and Transportation Officials (AASHTO), and Federal Highway Administration also provide guidelines for inspecting concrete infrastructure. Sample guidelines for inspecting concrete structures are summarized in Table 8. For example, in the ACI 201.1R document, concrete defects are divided into three major categories including cracking, deterioration (distress), textural features, and phenomena relative to their development. Table 9 shows a summary of defect types in each major category. To ensure the brevity of the paper, a detailed definition of these defect terminologies is not presented herein. Readers can refer to the ACI 201.1R document for more details. It can be concluded that each major category is subdivided into numerous specific defect types with different causes, which may lead to various consequences.

**Table 8:** Sample inspection guidelines for concrete and masonry structures

Concrete	Masonry
ACI 201.1R, “Guide for Conducting a Visual Inspection of Concrete in Service” ACI 207.3R, “Practices for Evaluation of Concrete in Existing Massive Structures for Service Conditions” ACI 224.1R, “Causes, Evaluation, and Repair of Cracks in Concrete Structures” ACI 311.1R, “ACI Manual of Concrete Inspection (SP-2)” ACI 364.1R, “Guide for Evaluation of Concrete Structures Before Rehabilitation” AASHTO, “Guide Specifications for Strength Evaluation of Existing Steel and Concrete Bridges”	The US Masonry Society, “Masonry Inspection Checklist” The US Masonry Society, “TMS 402/602-16 Building Code Requirements and Specification for Masonry Structures” NYC Building Code, “Structural Tests and Special Inspections” Snohomish County Code, “Residential Code - Frame and Masonry Inspection” Canadian Concrete Masonry Producers Association (CCMPA), “Concrete masonry inspection (NCMA)” Province of Manitoba, Canada, “Heritage Building Maintenance Manual”

**Table 9:** A short summary of visual defect types in ACI 201.1R

Major categories	Cracking	Deterioration	Textural features and phenomena relative to their development
Detailed defect types	Craze cracks, D-cracks, diagonal crack, hairline cracks, longitudinal cracks, map cracking, pattern cracking (with a repeated sequence), shrinkage cracking, plastic shrinkage cracking, temperature cracks, random cracks, transverse cracks	Chalking, curling, deflection/deformation/distortion, delamination, disintegration, drummy area, dusting, efflorescence, exfoliation, exudation, joint deficiencies, joint spall, joint sealant failure, joint leakage, joint fault, leakage, mortar flaking, peeling, pitting, popout, scaling, spalling	Air void, blistering, bugholes, cold joint, cold-joint lines, discoloration, honeycomb, incrustation, laitance, sand pocket, sand streak, segregation, staining, stalactite, stalagmite, stratification
Quantification metrics	Crack width, crack patterns, etc.	Spalling surface dimensions, spalling depth, popout diameter, depth of surface mortar loss, etc.	Air void diameter, bug hole diameter, etc.

On the other hand, masonry materials are widely seen in historic structures, and more recently used as concrete masonry in numerous modern projects to construct residential, commercial, and institutional buildings, due to their benefits such as high strength, great economy, and high fire resistance, etc. Inspection of masonry structures may cover a wide range of aspects such as the mason units, mortar, grout, reinforcement, connections, weather and water-resistive effects, and other serviceability aspects, etc. Structural failure-related damages include cracks, spalling, efflorescence, large deformation of reinforcing steel (in the case of reinforced masonry), connection failure, etc. Sample existing inspection guidelines for masonry structures are summarized in Table 8.

Correspondingly, the technology gap and challenges can be summarized. A screening of research papers published between 2018 and 2023 indicates most recent development or adoption of CV methods for concrete and masonry damage evaluation are focused on crack, and some on spalling, efflorescence, and steel reinforcement damage. In comparison, it can be expected that a well-trained and experienced inspector is able to identify all concrete defect types shown in Table 9, many of which are not addressed by existing CV methods. Most existing vision methods simply localize cracks with bounding boxes or pixel masks, without further distinguishing crack types (e.g., Crazing cracks, D-cracks, diagonal cracks). As different cracks have different causes and are related to different failure modes, they should be identified early to avoid catastrophic events. For example, diagonal cracks at about 45 degrees to the structural member are typically caused by shear stress. In this case, to automatically identify them as diagonal cracks, it is required to develop more advanced vision methods that can recognize both crack patterns and the structural member orientation concurrently. Furthermore, distinguishing between structural and non-structural cracks through CV methods or manual visual inspection is still challenging, even for experienced human inspectors. An effective way to differentiate between them is by referring to design drawings. If cracks are found on structural components, they can be treated as structural cracks. Otherwise, they are non-structural cracks. Therefore, the development of CV methods in conjunction with Building Information Modelling (BIM) systems which provide records of structural and nonstructural information (e.g., design drawings) is increasingly crucial.

### Existing Guidelines and Challenges: Steel Structures

Inspection of steel structures may broadly include component damage evaluation, surface paint evaluation, waterproof material inspection, etc. Some of the guidelines include: (1) AASHTO inspection manual; (2) American Society for Testing and Materials (ASTM) manual; (3) CSA S16, “Guidance for specifying third party inspection of steel structures”; (4) US Army Corps of Engineers, “Inspection, Evaluation, and Repair of Hydraulic Steel Structures”. With reference to these guidelines, steel cracks can be broadly classified as cracks due to many different factors such as fatigue, scratches, welding, and grinding. An experienced inspector is expected to identify all these crack types, not only based on crack patterns, but also on the surrounding indicators such as beach marks (which are concentric rings in a fatigue region), and ratchet marks (which are perpendicular to the origin of the fatigue crack). Fatigue cracks are the one of most critical types as they are closely related to structural performance. A review of the related research papers between 2018 and 2023 shows that existing related studies did not make further attempts to distinguish crack types. There exist many future research opportunities to tackle this problem by detecting cracks and surrounding indicators concurrently. For example, novel CV-based methods should be developed to recognize surrounding surface features such as beach marks, and used in combination with crack detection methods to make a final decision. Another potential CV-based solution is to use preinstalled cameras to conduct long-term monitoring to continuously track the crack growth process. Then, video analysis algorithms can be developed to evaluate the crack causes and extent.

On the other hand, existing guideline generally defines the steel corrosion damage extent with reference to the area of the corrosion portion relative to the normal portion on a steel surface. Consequently, existing CV-based methods have made great efforts in quantifying the steel corrosion area by segmenting the corrosion region in images and calculating the area of the segmented pixels. Despite the promising outcome, these methods generally require preprocessing of images, such as manual alignment of steel surface, and manual calibration of pixel dimensions to real-world dimensions. Future studies should be conducted to automate these processes, possibly by integrating the CV methods with other distance measurement devices.

As for the steel bolt loosening evaluation, it should be noted that steel bolts may get loosened without visually identifiable features such as bolt loosening rotation or exposed bolt thread. This is because steel materials may be subjected to fatigue, material softening, or plastic deformation, which causes bolts to lose the original clamping force and become loosened. It is very challenging for CV-based methods to capture these phenomena. Therefore, CV-based bolt loosening detection should be used as a complementary approach to other SDD solutions such as the torque testing method to inspect bolt loosening more reliably.

### Existing Guidelines and Challenges: Timber Structures

Inspection of structural timber components and systems may broadly include a variety of connections (threaded fasteners, concealed connections, adhesive anchors), staining and discoloration, insect activities-related damage, and wood component damage related to different failure modes due to significant environmental loads. Some of the sample inspection guidelines for timber structures include: (1) US Department of Agriculture, “Wood and Timber Condition Assessment Manual”; (2) Montana Department of Transportation, “Timber Bridge Inspection Guide”; (3) FP Innovations, “Technical Guide for the Design and Construction of Tall Wood Buildings in Canada - Chapter 9 Monitoring and Maintenance”; (4) US Army Corps of Engineers, “Inspection Procedures for Military Wood Structures”; (5) Illinois Building Code, “Special Inspections and Tests - Mass Timber Construction”.

Existing CV-based research on wood damage detection is focused on their natural defects, not structural damage caused by external loadings. Therefore, there exist numerous future research opportunities in this subdomain to develop CV-based methods for damage evaluation of various timber components such as beams, columns, walls, floor systems, etc. For example, typical structural damage and failure modes of the cross-laminated timber (CLT) components include: (1) local compression failure perpendicular to grain; (2) rolling shear failure; (3) interlaminated shear failure; (4) tensile fracture failure of parallel layer, (5) column penetration, etc. [189, 190]. The new CV-based methods for timber structures should comply with existing guidelines. For example, the Wood and Timber Condition Assessment Manual released by the US Department of Agriculture provides real-world inspection reports for real-world timber buildings and bridges on site, which can be informative and inspiring to researchers to develop CV-based methods to evaluate structural timber damage in real-world conditions.

### Selection of Codes, Standards, and Guidelines

Inspection of buildings and bridges must refer to local and national codes and standards relevant to the type of structures being inspected. These codes and standards often provide guidelines on structural safety, fire resistance, accessibility, and other critical aspects. These standards cover various aspects such as importance level of structures, damage criteria, inspection intervals, procedures, etc. During inspection, the age and type of the inspected structures may be considered. For example, older structures generally require more frequent inspections for issues related to aging materials, while inspection of modern buildings may target newer technologies and systems where their reliability and durability are more concerning. Past inspection and maintenance histories may be considered to guide the present selection of guidelines and perform the assessment accordingly. The occupancy and usage of the structures should also be considered when selecting inspection guidelines. For example, buildings such as hospitals and schools, and bridges with significant socio-economic consequences of collapse or traffic interruptions, may require more stringent and frequent inspections, with reference to a dedicated inspection guideline. In addition, external environmental conditions, such as extreme weather, seismic and strong wind activities, or corrosive environments, should also be considered when selecting and following a specific guideline suitable for that region.

### Summary and Additional Commentary

With the progressively enhanced SOTA model architecture, many CV models adopted in SDD have shown superior performance (e.g., ~100% in damage recognition and localization). The segmentation results, however, are presented as pixel coordinates (i.e., in the image space). To fully utilize these local segmentation results for engineering judgement, they should be associated with the specific structural members on which the damage appears. In other words, it is important to determine the damage location with respect to the local structural member, rather than just the pixel-wise locations expressed in the image coordinate system. For example, cracks formed in the mid-span of concrete beams have different causes and consequences, compared to cracks formed near beam-column joints. It is crucial for the CV methods to determine whether the cracks appear in the mid-span or near the two ends of beams to gain more in-depth knowledge of damage status during inspection.

On the other hand, despite the achievements of existing CV methods, the review of their compliance with structural inspection guidelines indicates that there still exists a big technological gap from what the CV methods are capable of to what a human inspector can. While images of structures are the only source for existing CV methods to make a judgment on structural status, a human inspector can establish a much more comprehensive chain of thoughts and reasoning with reference to the existing inspection guidelines. When a human inspector discovers poor conditions on site, the inspector should determine the root cause so that the problem can be addressed fundamentally. To illustrate this, an example is quoted from the Heritage Building Maintenance Manual as shown in Table 10. In the example shown, without an appropriate solution proposed using a chain of thoughts, the problem would reappear, because the fundamental issue, debris accumulation, was left unidentified and untreated.

**Table 10:** Manual inspection with a chain of thoughts and reasoning

Observation by a human inspector	Questions and conclusions raised with a chain of reasoning
A cornice falls off	Why did the cornice fall off? It was weak and the ice pulled it down. Why was it weak? Excessive moisture and decay caused the weakness. Where was the moisture? It was found in the cornice and the gutter. Why was the moisture in the gutter? Debris buildup trapped it there. Where did the debris come from? Trees overhanging the roof. In conclusion, the root cause is the debris accumulation. The debris should be cleaned or the trees should be dealt with to address the issue fundamentally.

In most existing related studies, unimodal CV methods were developed, which analyzed structural damage conditions based on image input only. However, a more critical question is how to develop more advance machine learning models that can automatically read professional codes and guidelines, analyze collected information and identify damage, and eventually generate professional inspection reports, which can more effectively enhance the current manual inspection practice. The generated inspection reports should follow a standardized format defined by inspection guidelines, including not only the local component defects, but also the global condition rating, causes of defects, recommended repair strategies, etc. By scrutinizing the existing inspection guidelines, it can be concluded that embedding the contents of inspection guidelines into the CV models (so as to establish a guideline-informed CV method) will provide a much more useful solution to enhance the existing inspection practice. However, this is very challenging and almost impossible for existing unimodal CV models, because only visual information (i.e., pixel values) is involved in unimodal CV methods. In comparison, human inspectors have enriched engineering theory knowledge and extensive field experience. During inspection, they can read inspection guidelines, capture damage information from visual perception, use specialized sensors or equipment to get additional measurements, making them much more knowledgeable than using CV methods alone. Therefore, the authors would argue that there exists a fundamental gap between the capability of existing unimodal CV methods to what human inspectors can do. It can be concluded that existing unimodal CV methods cannot replace human inspectors on its own, but instead, should be further enhanced and used as a tool with many other advanced sensing technologies together to provide more comprehensive inspection solutions. This leads to further discussion on future opportunities in Sect. 6.

## Challenges, Ongoing Work, and Future Research Opportunities

### Future Research Direction 1: Advanced Multimodal CV Algorithms

It is noted that earlier CV methods were primarily focused on qualitative assessment such as damage classification, and progressively transitioned to more quantitative assessment such as localization and quantification. To enhance the accuracy and usefulness of the localization and quantification process, CV research has been shifted from 2D vision-based evaluation to 3D vision-based evaluation. Nevertheless, the applicability of existing CV methods is still quite limited to certain structural component types, damage types, and failure modes, etc. For example, novel vision-based SDD methods should be further expanded for all structural categories, particularly for timber structures which are almost unexplored. Kodytek et al. [191] presented a dataset of wood surface defects, which may be a starting point for training and validation of data-driven CV algorithms for timber damage detection. Similarly, apart from common concrete damage types (e.g., cracks, spalling, etc.), novel CV algorithms should be developed to address other critical damage types of concrete structures (e.g., honeycombs, moisture spots, etc.) [192]. Furthermore, existing CV-based SDD methods using attention mechanisms and Transformer architectures are more limited than CNN-based methods. Research has shown CV methods with attention mechanisms can yield excellent performance in dealing with complex damage detection scenarios. For example, Gao et al. [60] have shown that the Transformer-based CV methods, when combined with multi-task learning, can effectively tackle multi-attribute and multi-tasks for SDD problems. Ruggieri et al. [192] developed an enhanced version of YOLO variant by incorporating attention mechanisms to enable the model to target the most related details in an image so as to improve the performance of the model. These studies were primarily validated on concrete structures. In the future, a similar concept should be further investigated in various damage detection scenarios.

**Fig. 7:**
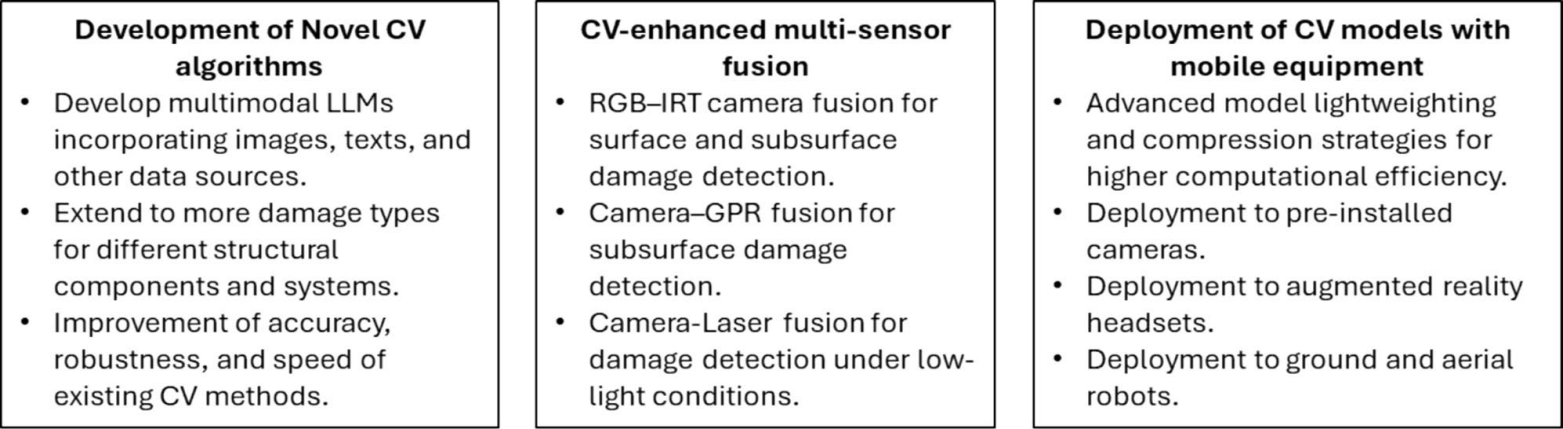
Future research directions

More importantly, as discussed in Sects. 4 and 5, most existing related research is generally limited to unimodal CV methods, where the models can only generate low-level outcomes such as image classification labels, bounding boxes, and pixel masks. In contrast, in current inspection practices, human inspectors need to write professional inspection reports expressed in natural language along with necessary photos taken on-site to record structural damage and potential hazards, with reference to specific inspection guidelines. There exists a large scientific and technological gap in automatically converting the image-level predictions into more meaningful outcomes such as inspection reports, which will better comply with existing inspection practices and be more useful to decision-makers. In principle, it is very challenging for existing unimodal CV methods to outperform human inspectors, because the unimodal CV methods take visual information as a single source input, while human inspectors can read codes and guidelines, capture information from the visual perception, with rich engineering theory knowledge, years of field experience, and many other specialized measurement tools to aid the inspection process. Fortunately, recent breakthroughs by multimodal Large Language Models (LLMs), such as GPT and DeepSeek, open numerous future possibilities to build multi-tasking AI agents in dealing with data sourced from multiple domains concurrently. For example, these multimodal LLMs are capable of inputting images, texts, sound waves, etc., and generate a more comprehensive prediction. Within the context of CV-based SDD, advanced multimodal Vision-Language Models can be developed in the future, which can automatically read codes and guidelines, process images, and generate inspection reports expressed in natural language, in an end-to-end manner. In a broader context, based on the promising results achieved by the prevalent ChatGPT and DeepSeek recently, it can be expected that the future LLMs trained on a wide variety of damage sensor data have a great potential to effectively take multi-sensor data as inputs and perform multi-objective assessments in the area of SHM. These kinds of generative AI models can be trained on a broad range of data sources, which has great potential to establish a chain of thoughts and reasoning as human inspectors to partially or eventually fully replace manual inspection in the future. Currently, training LLMs on multi-source large datasets is very expensive, which is mainly conducted by giant tech companies such as Microsoft and Google. The cost of training generally goes down over time, and the potential of these large AI models cannot be overstated. On the other hand, small datasets may be used at an early stage for proof-of-concept and gradually enhanced with more data available. For example, very few recent studies investigated the capability of multimodal Vision-Language Models for structural condition assessment [193, 194]. Although trained on small datasets, the models have shown a great potential to generate assessment results including images and descriptive texts expressed in natural language. The capability of these models can be further explored in automatic generation of structural inspection reports, using the same way as professional human inspectors do. Figure 7 summarizes future research directions.

### Future Research Direction 2: CV-Enhanced Multi-Sensor Fusion

It has been recognized by many existing studies that CV-based SDD methods are not intended to replace other SHM methods (e.g., vibration-based methods, NDTE methods). Instead, it will act as a complementary solution to these methods to achieve a more comprehensive inspection of civil infrastructure. Besides, human inspections generally carry multiple equipment and measuring tools during inspection. Within this context, fusing the image-based approach with other sensing technologies will enhance the capability of inspection. Below are a few potential research directions including the authors’ ongoing work.

An RGB camera is generally used to capture surface damage of structures. However, sub-surface damage (e.g., concrete delamination) can also be concerning. In this case, the Infrared Thermography (IRT), and Ground Penetrating Radar (GPR) technologies may be used to detect subsurface voids. Integration of these sensor devices with RGB cameras will allow both surface and subsurface damage detection. For example, Kuchipudi et al. [195] explored the use of GPR as a reliable non-destructive tool for structural concrete inspections. The study emphasized the potential of GPR, combined with advanced CV methods and deep neural networks, for detecting anomalies within reinforced concrete elements. The research identified several challenges and opportunities in the current GPR-based manual detection models and their transition toward automation aided by CV methods. It was suggested that integrating current post-processing techniques with state-of-the-art deep learning models can make GPR a more mainstream tool for field inspections, with potential improvements in accuracy and efficiency.

On the other hand, many existing CV-based algorithms were developed in the area of 2D computer vision (i.e., processing 2D images), which generally requires human intervention to convert the damage evaluation outcomes from 2D space to real-world 3D space to quantify the damage accurately. Some recent studies utilized 3D vision methods to address this issue by reconstructing 3D point clouds of structural components, which are accurate but relatively time-consuming, hindering its deployment as a rapid or real-time inspection method. Future research should be investigated by integrating camera and laser scanners for more balanced 3D reconstruction (i.e., accurate enough while being efficient), leveraging the complementary nature of the two sensor types [196]. For example, Yan et al. [197] made an early attempt at the integration of image and LiDAR data to detect and quantify concrete cracks. With a high-resolution camera and high-end LiDAR scanner, the study showed an average of 85% quantification accuracy. Hua et al. [198] developed a 3D reconstruction system for detecting concrete defects using optical laser triangulation, combined with a sports camera and a linear laser ray generator. The system addresses two primary imaging errors found in traditional methods: distortion from reflectivity and occluded light paths. The proposed spacetime analysis-based scanner showed enhanced capability in 3D defect reconstruction, especially in capturing depth information, vital for assessing defect severity. Chow et al. [133] proposed a mobile data collection system, integrating an advanced 360° camera and Light Detection and Ranging (LiDAR) device to obtain images and 3D point clouds concurrently. Using the simultaneous localization and mapping (SLAM) algorithm, the 3D site reconstruction can be done with the LiDAR data. Meanwhile, the image data can be processed by machine learning methods such as CNN to evaluate structural defects which can be mapped to respective structural elements due to the integrated nature of these devices. More recently, Li et al. [199] have proposed a simple yet effective inspection setup that integrates low-cost laser devices and cameras to quantify concrete defects, particularly on non-texture surfaces which cannot be reconstructed by traditional camera systems.

Furthermore, to process the multi-source measurement data acquired from the sensor fusion (e.g., cameras, laser devices, ultrasound sensors, ground penetrating radars, etc.), it may be rational to develop more advanced unified algorithms (e.g., integrating the capability of signal processing, image processing, and point cloud processing in a single pipeline) to process these multi-source data recorded in different formats. To achieve this, multimodal machine learning algorithms with multi-task learning capabilities can be further explored in their accuracy, robustness, and efficiency in detecting different damage types.

### Future Research Direction 3: Deployment of CV Methods with Mobile Equipment

To make a larger impact on real-world engineering practices, CV methods should be deployed to mobile equipment and edge computing devices, such as pre-installed cameras, augmented reality headsets, and mobile aerial and ground robots. For model deployment, advanced lightweighting and compression strategies that do not significantly degrade model performance should be further explored for novel CV methods, such as pruning, knowledge distillation, and quantization. These optimization methods will enable the model to run quickly on mobile or edge computing devices with limited computational resources, which can satisfy the needs for rapid or real-time on-site inspection.

To fully automate the inspection practice, it is crucial to take one leap forward by empowering mobile equipment with advanced CV methods [200]. For example, recent research involving CV-aided robotic technologies inspection has gained great traction for inspection of buildings [29, 132, 166, 201], bridges [202–204], and other civil infrastructure [205]. In general, these studies made early attempts to partially automate some processes during inspection but have not achieved fully autonomous navigation and inspection by robots for the whole structure. Each civil infrastructure is unique and can have very different geometry and component layouts, which means the navigation and inspection algorithms developed in one scenario can be difficult or even impossible to generalize to the other. This makes fully autonomous navigation and inspection a very challenging problem. As a result, existing development in this subdomain remains at the infancy stage, which opens avenues for numerous future opportunities.

Within this context, while preinstalled cameras (e.g., security cameras) may be programmed as long-term damage monitoring devices (e.g., track potential crack growth, bolt loosening, etc.), on the other hand, robots equipped with a variety of sensors should also be programmed and further developed for automated inspection tasks. Unmanned ground robots (UGV) are generally classified as wheeled robots, legged robots, or hybrid robots (i.e., with wheels on the legs). These robots should be selected for different inspection scenarios. For example, legged UGVs (dual-legged, quadruped, spider-like robots) can be considered for inspection across uneven terrains in tunnels or post-disaster sites, or inside buildings where the robots are required to climb staircases. The wheeled UGVs can be considered for road or bridge deck surface inspection as they can travel more efficiently at more economical power consumption than legged UGVs. Unmanned aerial vehicles (UAV) can be considered for high-altitude inspection, such as high-rise buildings and bridges. Further, as civil infrastructure is large and complex in general, it is inefficient to apply a single robot for the inspection of a large-scale infrastructure. Future studies should be conducted to explore multi-robot collaboration schemes (e.g., drone fleet, UAV–UGV collaboration) to enhance the efficiency and capability of inspection. In all cases, the CV methods can be used to assist the autonomous navigation and damage evaluation process.

## Conclusions

This paper provides a systematic review of recent advances in CV methods for structural damage evaluation by discussing recent articles published during the past five years. Based on their applicability, the CV algorithms are first classified into damage recognition, localization, and quantification, where the theory, architecture, and progressive improvements of the algorithms have been reviewed in detail. Then, applications of these CV algorithms for real engineering practices are discussed and compared with the existing inspection guidelines to identify key challenges and technological gaps. Finally, based on the identified challenges and ongoing work, the research concluded three major future research directions for CV-based SDD including:

1. Development of new CV methods that can address various types of structural components and systems in different categories. New SOTA model architecture may be further explored in different damage evaluation scenarios. Meanwhile, the research should continue to make a transition from 2D vision to 3D vision to provide more practical evaluation outcomes. Further, the research should potentially leverage the technology advancement of large AI models to establish a chain of thoughts during inspection like what human inspectors can do.
2. Integration of CV methods with other sensing technologies to facilitate a more comprehensive periodic inspection, postdisaster inspection, and long-term monitoring solutions. Sensor fusion strategy should aim to enhance the capability of monitoring from a single type of sensors, and achieve a more balanced solution between efficiency, accuracy, and cost. Advanced unified algorithms may be developed that can process different types of sensor data in a single framework (e.g., signal processing, image processing, and point cloud processing)
3. Implementation of CV methods with mobile equipment towards fully autonomous inspection in various scenarios, such as routine inspection and post-disaster inspection. Future research such as investigating different types of sensors on robots to enable multi-data collection, developing autonomous navigation and data collection methods for robots to adapt to different inspection environments, and developing collaborative multi-robot schemes, may be further explored.

In addition, the limitations of the present study are summarized herein. First, the paper is focused on the infrastructure in the area of structural engineering such as buildings and bridges. The infrastructure in geotechnical and transportation engineering such as tunnels, dams and roads are excluded in the present study to limit the scope and maintain the review focus. Future review may be conducted to scrutinize the gap between the capability of the current CV methods and their respective inspection guidelines. Secondly, the paper is only focused on the use of CV for structural damage evaluation. It should be noted that CV methods have been successful in various structural engineering applications such as CV-based structural vibration measurement, CV-aided modal analysis, CV-aided structural experimental testing, etc. A review of recent advances in those subdomains may be provided in the future.

## Data Availability

No datasets were generated or analysed during the current study.
